# The Model Structures of the Complement Component 5a Receptor (C5aR) Bound to the Native and Engineered ^h^C5a

**DOI:** 10.1038/s41598-018-21290-4

**Published:** 2018-02-13

**Authors:** Amita Rani Sahoo, Richa Mishra, Soumendra Rana

**Affiliations:** Chemical Biology Laboratory, School of Basic Sciences, Indian Institute of Technology Bhubaneswar, Bhubaneswar, Odisha 752050 India

## Abstract

The interaction of ^h^C5a with C5aR, previously hypothesized to involve a “two-site” binding, (i) recognition of the bulk of ^h^C5a by the N-terminus (NT) of C5aR (“site1”), and (ii) recognition of C-terminus (CT) of ^h^C5a by the extra cellular surface (ECS) of the C5aR (“site2”). However, the pharmacological landscapes of such recognition sites are yet to be illuminated at atomistic resolution. In the context, unique model complexes of C5aR, harboring pharmacophores of diverse functionality at the “site2” has recently been described. The current study provides a rational illustration of the “two-site” binding paradigm in C5aR, by recruiting the native agonist ^h^C5a and engineered antagonist ^h^C5a(A8). The ^h^C5a-C5aR and ^h^C5a(A8)-C5aR complexes studied over 250 ns of molecular dynamics (MD) each in POPC bilayer illuminate the hallmark of activation mechanism in C5aR. The intermolecular interactions in the model complexes are well supported by the molecular mechanics Poisson–Boltzmann surface area (MM-PBSA) based binding free energy calculation, strongly correlating with the reported mutational studies. Exemplified in two unique and contrasting molecular complexes, the study provides an exceptional understanding of the pharmacological divergence observed in C5aR, which will certainly be useful for search and optimization of new generation “neutraligands” targeting the ^h^C5a-C5aR interaction.

## Introduction

Complement component fragment 5a receptor (C5aR) is one among the two chemoattractant receptors known in the rhodopsin family of G-protein coupled receptors (GPCR)^1^. C5aR is known to be stimulated by the ^h^C5a^2^, one of the most potent inflammatory modulator of the complement system, driving the host-defense mechanism. However, the protecting shield is often weakened or lost due to the aberrant stimulation of C5aR, exposing the host to variety of inflammatory, autoimmune and neurological disorders^3,4^. Though, understanding the ^h^C5a-C5aR interaction for therapeutic intervention appears lucrative, clinical breakthroughs remains largely limited, apparently due to the lack of atomistic understanding of the molecular interactions, between the ^h^C5a and C5aR. Thus, for realizing better and improved complement therapeutics for future clinical practices, it is highly imperative to obtain a rational picture of the molecular complexation between ^h^C5a and C5aR, no matter how crude it may appear at this stage. Driven by large scale mutagenesis studies, the molecular complexation is hypothesized to involve two discrete sites^5^: (i) interaction between the NT peptide of C5aR with the bulk of ^h^C5a (site1) and (ii) interaction between the ECS of C5aR with the CT peptide of ^h^C5a (site2). It is apparently clear from the literature that the interactions at the “site1” play the anchorage function to arrest the ^h^C5a, whereas the interactions at the “site2” trigger the cellular responses of C5aR. Interestingly, such “two-site” binding paradigm has recently been structurally exemplified in few peptide or protein binding GPCRs of rhodopsin family^6,7^. Nevertheless, no such structural studies or refined molecular models illustrating the intermolecular interactions at both the “site1” and “site2” are currently available for ^h^C5a and C5aR.

In our quest to understand the ^h^C5a-C5aR interaction better, we recently generated unique structural models of C5aR^8^ and subsequently illustrated the plausible orthosteric “site2” on its ECS^9^, by recruiting a variety of functionally diverse small molecule ligands, including the CT peptide (^64^NISHKDMQLGR^74^) of ^h^C5a. In the current study, we subjected the modeled C5aR to pilot experimental scrutiny, involving biophysical techniques and further screened the model against the native agonist ^h^C5a^2^ (74 amino acids) and the engineered antagonist (73 amino acids) ^h^C5a(A8)^10^. Objective was to decipher the plausible orthosteric “site1” on the NMR derived NT peptide^11^, grafted to the modeled C5aR^9^ for generating the first set of distinct model molecular complexes, precisely illustrating the pharmaceutical landscape of the “two-site” binding paradigm in C5aR. Though, both ^h^C5a and ^h^C5a(A8) share ~90% sequence identity, ^h^C5a(A8) competitively binds to the C5aR, albeit weakly (IC_50_ ~ 35 nM) compared to ^h^C5a (IC_50_ ~ 3 nM) for reasons clearly not described^12^. Structurally ^h^C5a(A8) appears to be an allosteric conformer of ^h^C5a, that imparts the antagonistic effect on C5aR, due to its engineered CT (^64^NISFKRSLLR^73^) sequence. Interestingly, several single point mutations on the CT of ^h^C5a(A8) has also been described that can reverse the antagonism of ^h^C5a(A8) to agonism^12^. However, the mechanism of such action is still unclear in structural terms. In continuation to our earlier reports^8,9,13^, the comparison of the ^h^C5a-C5aR, ^h^C5a(A8)-C5aR model structural complexes, including the CT peptide variants of ^h^C5a(A8) presented in the study provide the necessary rationalization important for understanding the observed antagonism and the switching of antagonism to agonism at the “site2” of C5aR. Moreover, the native agonist (^h^C5a-C5aR) and the engineered antagonist (^h^C5a(A8)-C5aR) bound model complexes, respectively presented in the current study rationalize a large set of point mutation based binding and signaling data^12,14–20^, by estimating the residue specific energetic contribution toward overall binding in structural terms. The model complexes, thus appear as a useful template for structure-based drug design, by illuminating the intermolecular interactions at atomistic resolution, highly essential for modeling and discovery of potential disruptive pharmacophores targeting the ^h^C5a-C5aR interactions.

## Results

### Validating the model structure of C5aR

**Figure 1:**
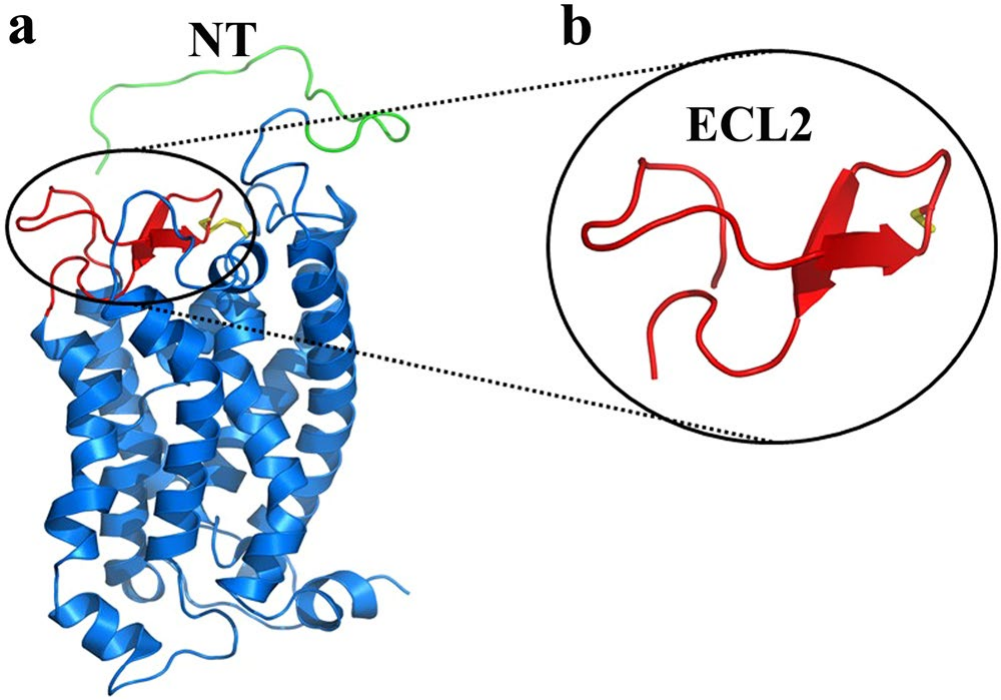
(**a**) The model structure of C5aR illustrating the probable structure of ECL2 peptide (red) and the NMR derived structure of NT peptide (green). The conserved ECL2-TM3 disulfide bond is also highlighted in yellow. (**b**) The highly twisted short stranded β-sheet conformation of the ECL2 peptide derived from the in silico folding studies, illustrating the C188 in the loop region.

**Figure 2:**
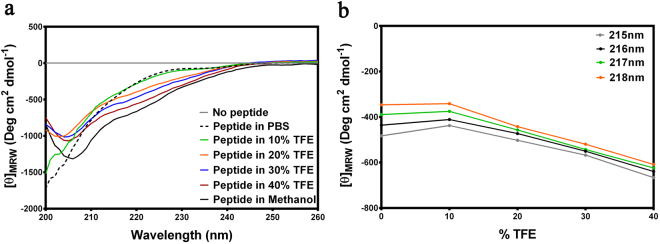
(**a**) Signature CD spectra demonstrating the effect of different solvent conditions on the secondary structure of the ECL2 peptide. (**b**) Effect of TFE concentration on the CD intensities in the 215–218 nm β-sheet region.

The topologically unique model of C5aR described earlier^8,9^, presented in Fig. 1 illustrates a modestly folded β-hairpin like structure with ~30% residues in ordered β-sheet conformation, as estimated from the in silico folding studies of the predicted extended extracellular loop 2 (ECL2) polypeptide [Ac-Y174-RVVREEYFPPKVL**C188**GVDYSHDKR-R198-NH_2_]^8^. The C5aR model (Fig. 1) also feature an unordered NT peptide, mostly derived from the previously reported NMR studies^11^. Given the known structure of many GPCRs^8^, it is highly unlikely that individual transmembrane (TM) domains of C5aR will demonstrate a structure other than α-helix, though their topological arrangement as a 7 TM bundle may slightly vary in real experimental conditions from the modeled C5aR (Fig. 1), which is a matter of future detailed structural studies. Further, structural analysis of the loop structures in known GPCRs evidence that the ECL2 peptide is longest among all other loops, and demonstrates structural diversity^8^. Thus, we decided to probe the conformational state of the predicted ECL2 peptide in various solvent conditions using circular dichroism (CD) and ^1^H-NMR spectroscopy. The ECL2 peptide was synthesized over solid phase with C188/S to avoid the unwanted aggregation in solution. Further, serine being isostructural to cysteine may not drastically alter the possible conformation of the ECL2 peptide in solution. Interestingly, in agreement with our folding simulation studies reported for the extended ECL2 peptide^8^, the synthetic ECL2 peptide with ≥95% purity (Fig. S1) demonstrated a CD signature (Fig. 2) reminiscent of a highly twisted short-stranded β-sheet conformation (Fig. 1b), frequently observed for β_II_-class of proteins^21^. Addition of 10–40% trifluoroethanol (TFE), a hydrogen bond promoting solvent^22^ to the PBS buffer, did not alter the overall CD signature but enhanced the intensity of the observed CD signature of the ECL2 peptide. Even in 100% methanol, the peptide demonstrated a similar CD signature with highest intensity, indicating the role of solvent dielectric on the overall conformation of the ECL2 peptide^22^. A detailed comparison of the CD intensities at 215–218 nm, 222 nm and 208 nm indicated that addition of TFE perhaps enhances the % β-sheet content in the peptide (Fig. 2b). In support, estimation of [*θ*]_222_/[*θ*]_208_ provided a value of 0.65, indicating the presence of a 3_10_-turn^23^, and addition of TFE also did not change the estimated % helicity (~3%) further^24^. In further support to CD spectra, the ^1^H-NMR spectra of the ECL2 peptide in 10% D_2_O-water (Fig. S2) appeared well dispersed, indicating the presence of an ordered conformation of the peptide in solution. Nevertheless, the ECL2 being one of the major structural component in the ECS of C5aR that harbors the orthosteric “site2”, a separate detailed structural study can be undertaken later. Though inconclusive, prima facie, the pilot biophysical studies are in sync with the modeled conformation of the ECL2, which partially validate the presented model of C5aR (Fig. 1a), providing the necessary impetus to probe the “site1” on the modeled C5aR toward establishing a plausible “two site” binding interaction involving ^h^C5a and C5aR.

### Structural complex of ^h^C5a with C5aR

**Figure 3:**
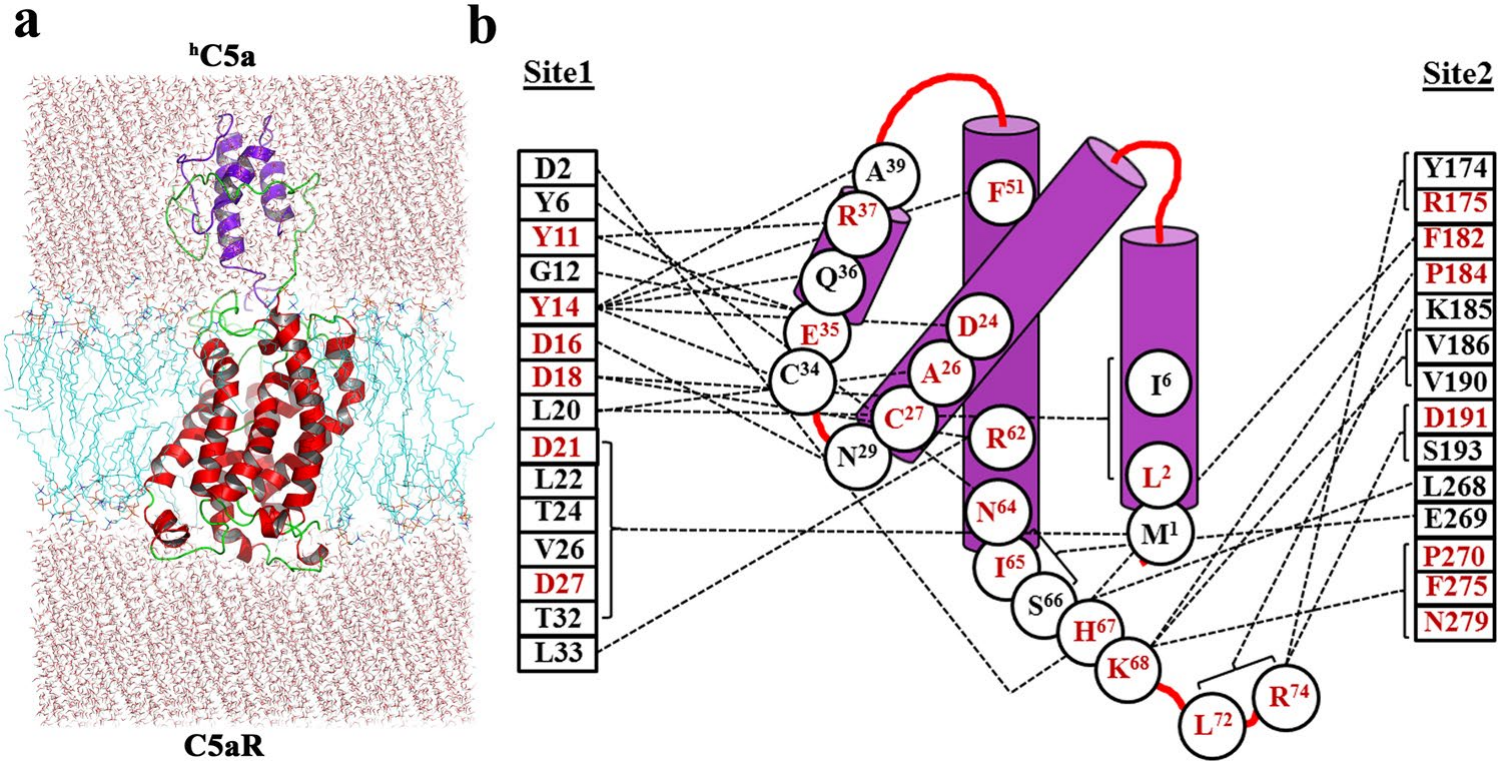
(**a**) Illustration of “two-site” binding in the model complex of ^h^C5a-C5aR inserted into the POPC bilayer. (**b**) Interaction map of ^h^C5a illustrating the “hot-spot” residues, respectively at the “site1” and “site2” of C5aR. “Hot-spot” residues that are known to modulate both binding and signaling upon mutation are highlighted in red.

**Figure 4:**
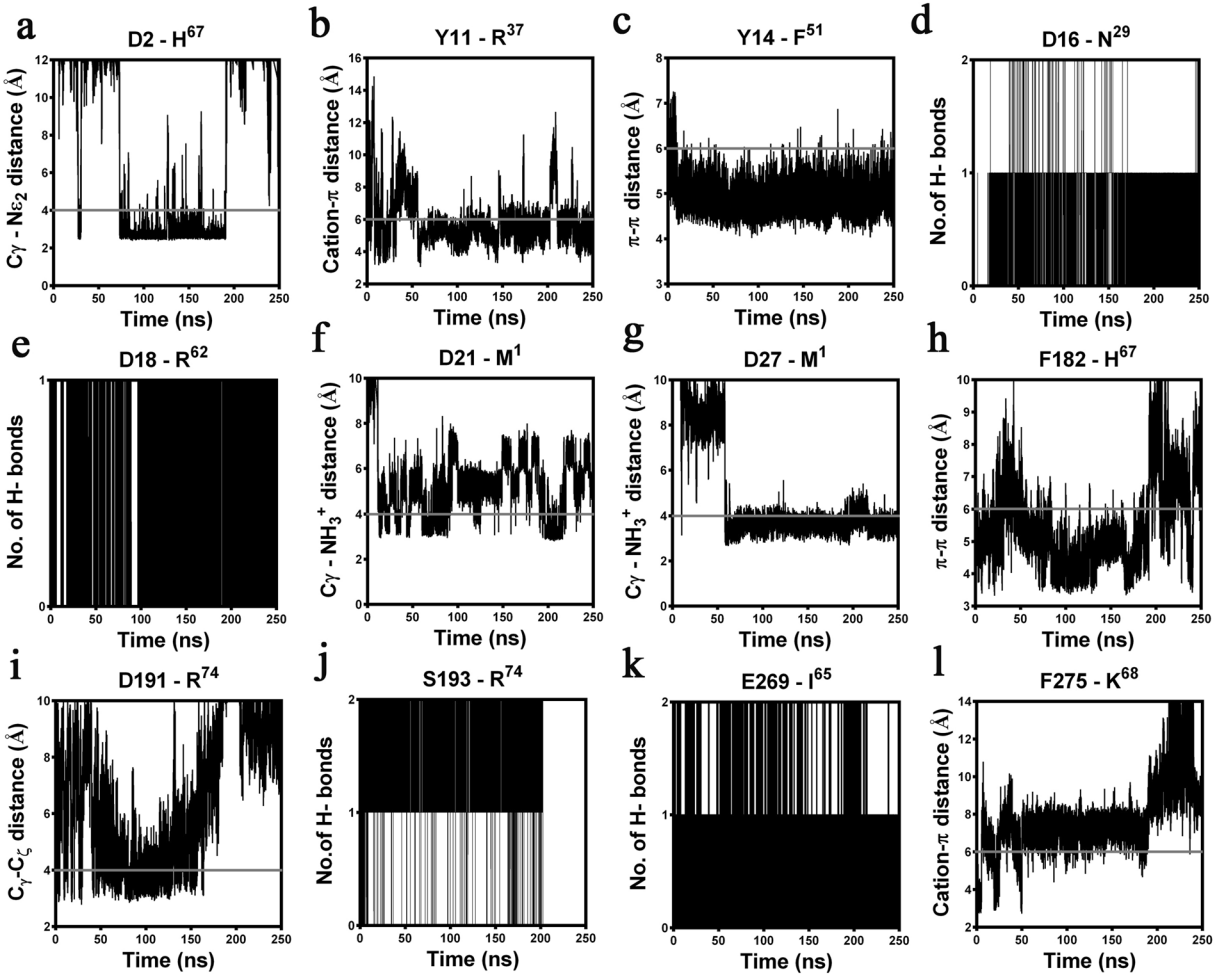
Summary of the specific intermolecular interactions monitored both at the “site1” and “site2” of ^h^C5a-C5aR complex over 250 ns of MD at 300 K in POPC bilayer. Interacting residues that are not superscripted represents C5aR and the residues that are superscripted represents ^h^C5a. The solid grey lines indicate the cut-off distance. (**a**) Moderate salt bridge interaction monitored between the D2 and H^67^. (**b**) Strong “cation-π” interaction observed between Y11 and R^37^. **(c**) Stable “π-π” interaction observed between Y14 and F^51^. (**d**) Strong hydrogen bonding noted between the side chain of D16 and the backbone NH of N^29^. (**e**) Strong hydrogen bond noted between the backbone carbonyl of D18 and the side chain NH of R^62^. (**f**) Stable salt bridge interactions observed between the terminal NH_3_^+^ of M^1^ and side chain of D21 and (**g**) D27. **(h**) Strong “π-π” interaction observed between F182 and H^67^. (**i**) Moderate salt bridge interaction noted between the side chain of D191 and the terminal CO_2_^−^ of R^74^. (**j**) Strong hydrogen bond between side chain of S193 and terminal CO_2_^−^ of R^74^. (**k**) Very strong hydrogen bond interaction between side chain of E269 and the backbone NH of I^65^. (**l**) Stable “cation-π” interaction observed between F275 and K^68^.

In our prior studies^9^, we have illustrated the interaction of ^h^C5a-CT peptide at the “site2” of the modeled C5aR with minimum interference from the NT peptide of C5aR. However, to illustrate a “two-site” binding interaction between ^h^C5a and C5aR, it is highly essential to understand the molecular interaction at the “site1” involving the bulk of ^h^C5a and the NT peptide of the C5aR. Thus, to decipher the molecular interaction at the “site1”, the NMR-derived NT-peptide^11^, grafted to the C5aR model [Ac-D2-SFNYTTPDYGHYDDKDTLDLNTPVD-K28-NH_2_] (Fig. 1a) was subjected to a sequential buildup docking studies combined with energy minimization against the most populated conformer of ^h^C5a, evolved over 50 ns of molecular dynamics (MD) study^13^. The docking protocol benchmarked against the CHIPS protein complex (Fig. S3) yielded an estimated K_i_ ~ 5.33 nM (−11.29 kcal/mol) for the best conformer of the NT peptide of C5aR complexed to ^h^C5a (Fig. S4), illuminating the most plausible “site1” on C5aR. The molecular complex gauged over 100 ns of MD studies appears to be stable in the explicit water at 300 K, suggesting that the modeled interactions depicted at the “site1” are physically viable (Fig. S5). Further, the most populated conformer of the “site1” complex evolved over the MD (Fig. S4) was subjected to structural assembly with the previously described C5aR complexed to ^h^C5a-CT^9^ at the “site2” for generating the complete ^h^C5a-C5aR complex (Figs 3a and S6). The resulting “two-site” binding structural complex of ^h^C5a-C5aR was carefully inserted into the POPC bilayer (Fig. 3a) as described^8,9^ and subjected to one quarter of a microsecond MD studies at 300 K. The “hot-spot” residues participating in variety of intermolecular interaction (Fig. 4) mainly hydrophobic, hydrogen bonding and salt bridge interactions (Fig. S7) at both the “site1” and “site2” of ^h^C5a-C5aR complex are schematically illustrated in the Fig. 3b. Sustainability of many such important residue specific intermolecular interactions at both “site1” and “site2” over the duration of MD are summarized in Fig. 4 (Fig. S7), indicating the physical viability of the interactions under experimental conditions, overall molecular stability and atomistic nature of the modeled ^h^C5a-C5aR complex.

### Structural complex of ^h^C5a(A8) with C5aR

The ^h^C5a(A8)^10^ is an engineered protein derived from ^h^C5a, which has been described to act as a potent antagonist (ID_50_ ~ 22 nM) against C5aR due to its engineered CT (^64^NISFKRSLLR^73^) sequence^12^. Interestingly the protein is also described to switch its function from antagonist to agonist by introducing a point mutation at R^69^ of its CT. Recent structural studies indicate that ^h^C5a(A8) is structurally different from native ^h^C5a (IC_50_ ~ 3 nM), and competitively bind weakly to C5aR (IC_50_ ~ 35 nM)^12^. Thus, we decided to probe the molecular interaction of ^h^C5a(A8) with C5aR, by subjecting the previously described C5aR model^8,9^ into action. Initially, we subjected the CT peptide of ^h^C5a(A8) and some of its variants to automated docking studies against the “site2” of C5aR, as described for ^h^C5a-CT^9^. Surprisingly, the A8, A8^Δ71–73^, and A8^R69D^ CT peptides of ^h^C5a(A8), respectively with an estimated K_i_ ~ 970 nM (−8.20 kcal/mol), K_i_ ~ 2.36 μM (−7.68 kcal/mol), and K_i_ ~ 117 μM (−5.36 kcal/mol), perfectly blocked the “site2” on C5aR (Fig. S8), surrounded by a cluster of hydrophobic residues. Further analysis revealed that the F^67^ on the CT peptide of both A8 and A8^Δ71–73^ is involved in a “π-π” interaction^25^ with F275 at the “site2” of the modeled C5aR (Fig. S9), as observed previously for PMX53 and NDT^9^. In contrast, the K^68^ on the CT peptide of A8^R69D^ demonstrated a similar “cation-π” interaction^26^ involving the F275 (Fig. S9), as observed previously for ^h^C5a-CT peptide^9^. Interestingly, the estimated affinity of the A8^R69D^ (K_i_ ~ 117 μM; −5.36 kcal/mol) CT peptide of the ^h^C5a(A8) is apparently in sync with our earlier estimation for ^h^C5a-CT peptide (K_i_ ~ 35 μM, −6.08 kcal/mol) that is known to demonstrate binding affinity of ~150 μM toward C5aR in PMNL membranes^27^. Interestingly, the interactions observed for the CT-peptide variants of ^h^C5a(A8) in the C5aR complexes, remained intact over 100 ns of MD (Fig. S9) in POPC bilayer, suggesting the physical viability of the modeled interactions and stability of the overall complex (Fig. S10).

**Figure 5:**
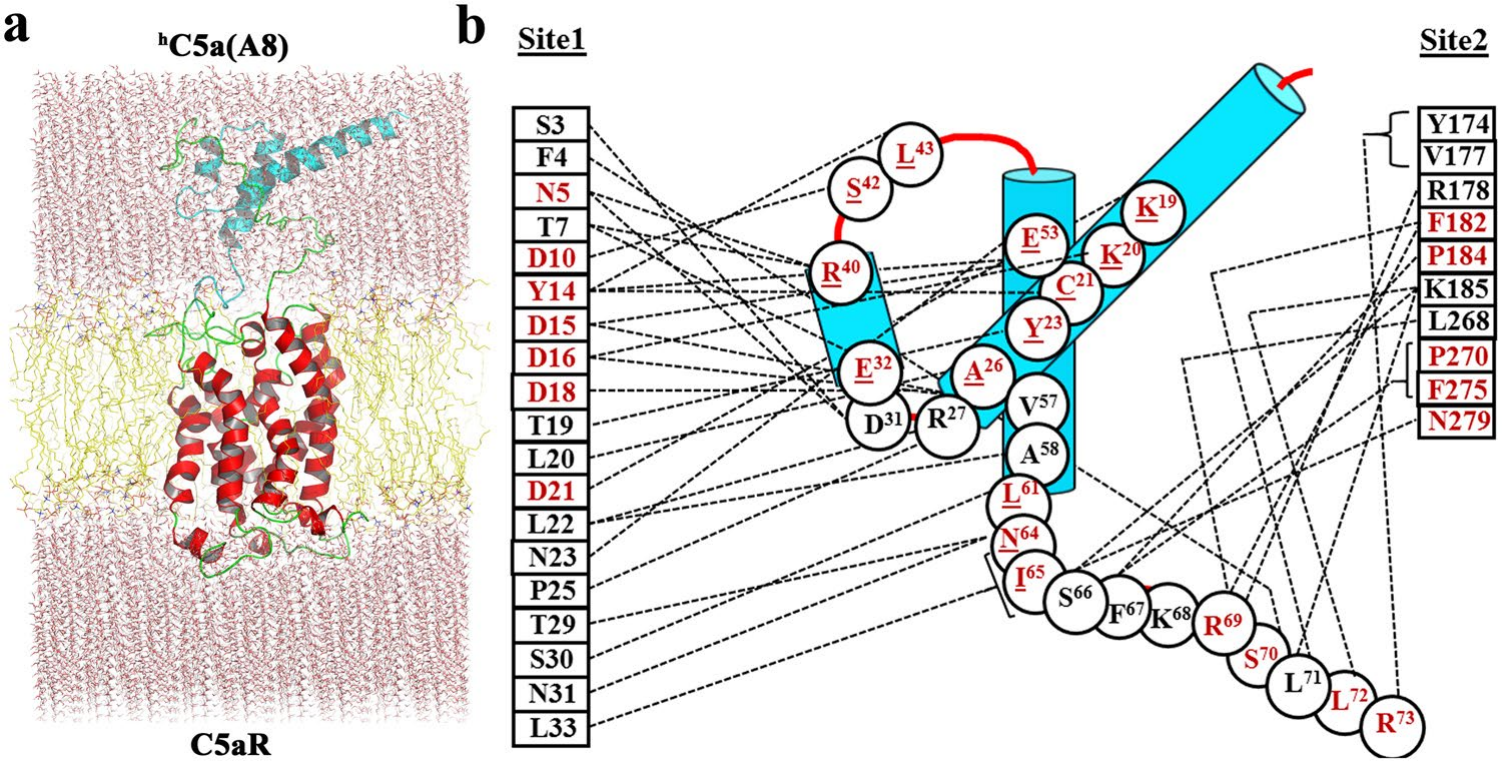
(**a**) Illustration of “two-site” binding in the model complex of ^h^C5a(A8)-C5aR inserted into the POPC bilayer. (**b**) Interaction map of ^h^C5a(A8) illustrating the “hot-spot” residues, respectively at the “site1” and “site2” of C5aR. “Hot-spot” residues that are known to modulate both binding and signaling upon mutation are highlighted in red. Residues of ^h^C5a(A8) whose mutation are known in ^h^C5a are shown in red and underlined.

**Figure 6:**
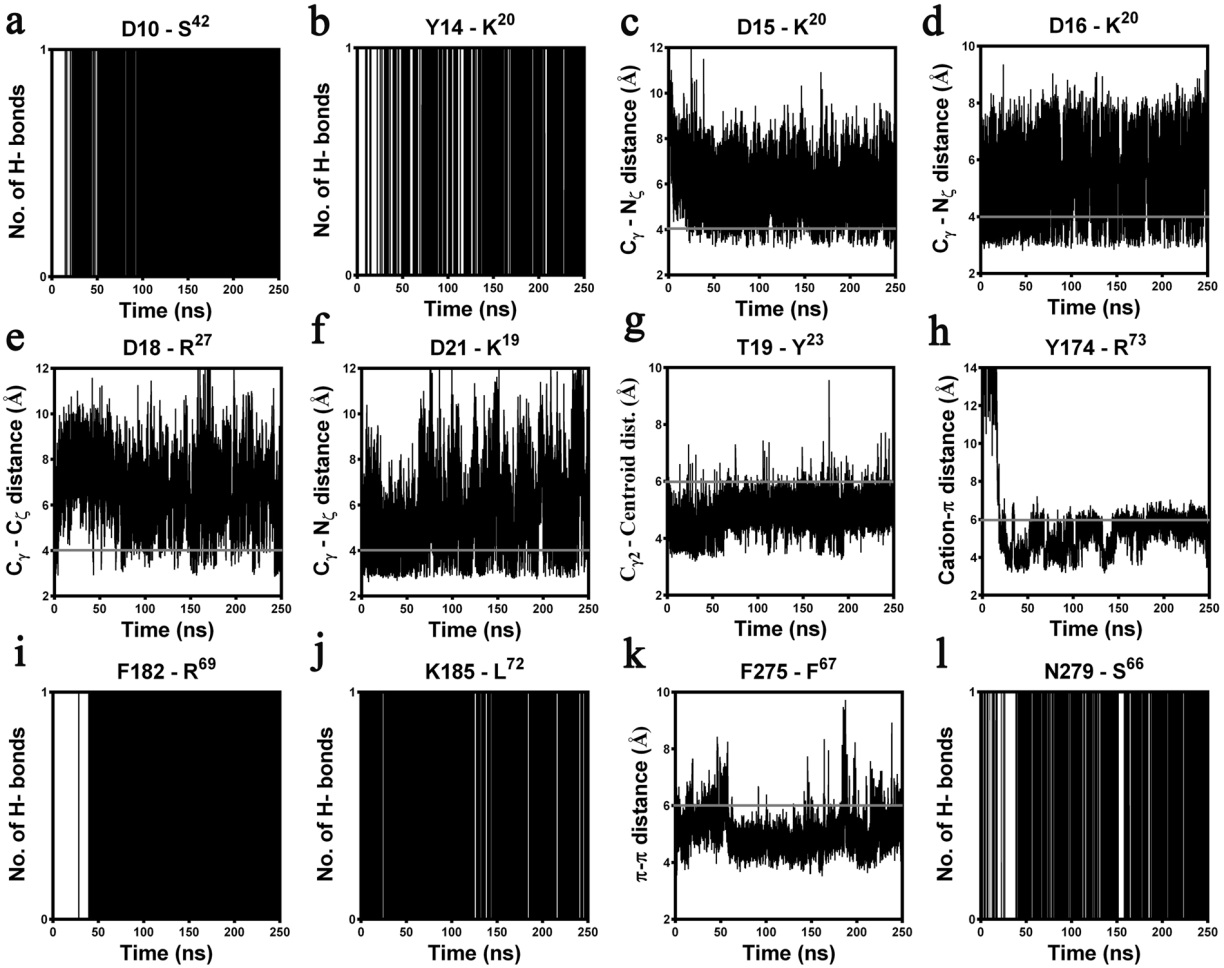
Summary of the specific intermolecular interactions monitored both at the “site1” and “site2” of ^h^C5a(A8)-C5aR complex over 250 ns of MD at 300 K in POPC bilayer. Interacting residues that are not superscripted represents C5aR and the residues that are superscripted represents ^h^C5a(A8). The distance cut-offs are shown as solid grey lines. (**a**) Very strong hydrogen bond interaction observed between the backbone CO of D10 and the backbone NH of S^42^. (**b**) Stable hydrogen bonding noted between the backbone CO of Y14 and the side chain of K^20^. (**c**) Stable salt bridge interactions observed between the head group of K^20^ and side chain of D15 and (**d**) D16. (**e**) Moderate salt bridge interaction observed between the side chain of D18 and R^27^ and (**f**) between the side chain of D21 and K^19^. (**g**) The strong hydrophobic interaction between Cγ_2_ of T19 and the centroid of Y^23^. (**h**) Stable “cation-π” interaction observed between the centroid of Y174 and the side chain of R^73^. (**i**) Strong hydrogen bonding noted between the backbone NH of F182 and the backbone CO of R^69^. (**j**) Very strong hydrogen bond interaction between the side chain of K185 and the backbone CO of L^72^. (**k**) Stable “π- π” interaction observed between F275 and F^67^. (**l**) Strong hydrogen bonding noted between the backbone CO of N279 and the side chain of S^66^.

As described for ^h^C5a, the NT-peptide of C5aR was also subjected to systematic stepwise docking against the ^h^C5a(A8), and the resultant complex illustrating the interaction at “site1” (Fig. S11) yielded an estimated K_i_ ~ 113 μM (−5.38 kcal/mol), compared to the interaction at “site1” for ^h^C5a (K_i_ ~ 5.33 nM; −11.29 kcal/mol). The observation is broadly in sync with experiments and can be attributed to overall structural difference between ^h^C5a and ^h^C5a(A8). The resultant molecular complex of ^h^C5a(A8) remained stable over 100 ns of MD in explicit water at 300 K, suggesting that the modeled interactions observed for bulk of ^h^C5a(A8) at the “site1” of C5aR are physically viable (Fig. S12). Further, by applying requisite geometrical constraints, the ^h^C5a(A8) complexed to NT-peptide of C5aR (site1; Fig. S11) was subjected to structural assembly with the C5aR complexed to CT peptide of ^h^C5a(A8) at the “site2” (Fig. S10) for generating the complete ^h^C5a(A8)-C5aR complex (Figs 5a and S13). The modeled complex was further subjected to MD studies in POPC bilayer (Fig. 5a) at 300 K over one quarter of a microsecond. The “hot-spot” residues involved in the “two-site” binding interaction between ^h^C5a(A8) and C5aR are schematically illustrated in Fig. 5b. The various intermolecular interactions observed between the hot-spot residues of the complex are also sustained over the duration of MD (Figs 6 and S14), indicating the overall stability of the complex at par with the ^h^C5a-C5aR complex.

### Estimation of the energetic contribution of “hot-spot” residues

**Figure 7:**
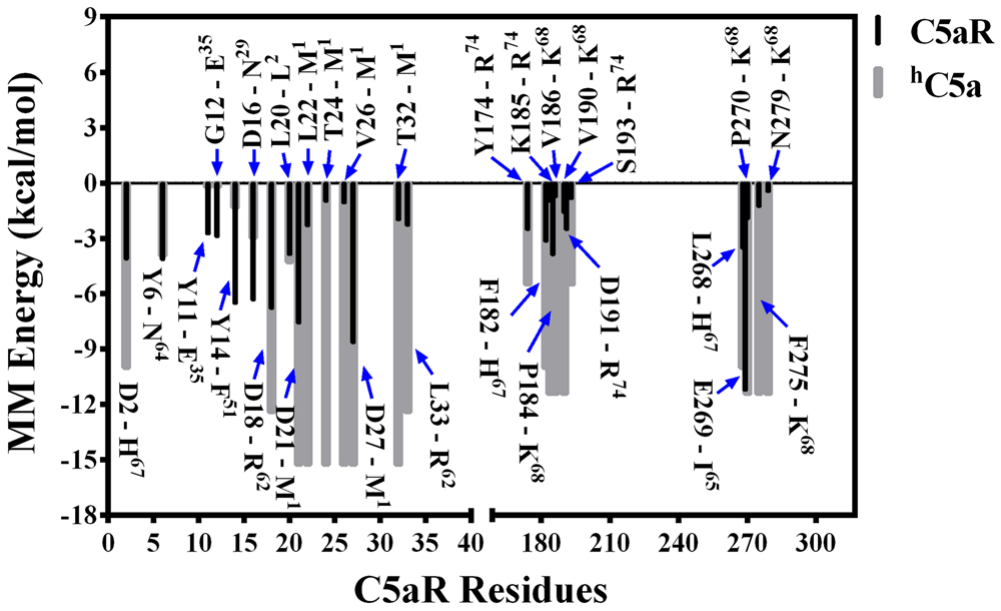
Decomposition of the MM energy of “hot-spot” residues contributing toward overall binding free energy of ^h^C5a-C5aR complex. Energetic contribution of ^h^C5a and C5aR residues are respectively shown in grey and black. ^h^C5a residues are indicated with superscripts.

**Figure 8:**
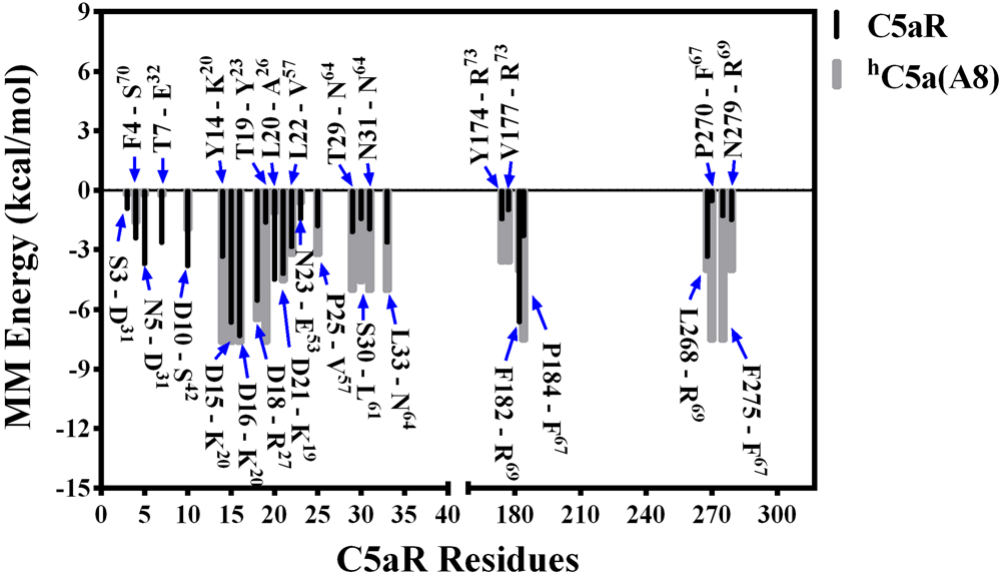
Decomposition of the MM energy of “hot-spot” residues contributing toward overall binding free energy of ^h^C5a(A8)-C5aR complex. Energetic contribution of ^h^C5a(A8) and C5aR residues are respectively shown in grey and black. ^h^C5a(A8) residues are indicated with superscripts.

The molecular mechanics Poisson-Boltzmann surface area (MM-PBSA/MM-GBSA) has been a useful tool for estimating binding free energies of various protein-ligand complexes^28–30^ in remarkable correlation with the experimental results^31,32^, though application of MM-PBSA calculation to membrane proteins still remains tricky for various reasons^33^. Nevertheless, we decided to recruit the method for estimating an apparent binding energy of ^h^C5a/^h^C5a(A8)-C5aR complexes in a moderate dielectric medium, by randomly selecting 150 conformers each from the most populated cluster (Fig. S15), evolved over the duration of the respective MD trajectories. This decision of modulating the dielectric was somehow influenced from our CD studies on ECL2 peptide in different solvent gradients. As presented in Fig. 3 (Fig. S6) and Fig. 5 (Fig. S13), the binding of ^h^C5a or ^h^C5a(A8) is largely influenced by the solvent exposed ECS and NT residues of C5aR. Thus, to avoid the complexity of the overall calculation involving the lipid bilayer, only the interacting residue pairs from C5aR and ^h^C5a/^h^C5a(A8) were subjected to MM-PBSA calculations, respectively for estimating the binding free energy. Under such conditions, the ^h^C5a-C5aR complex provided an estimated average binding energy of ~−16.12 ± 4.2 kcal/mol (K_i_ ~ 1.65 × 10^−12^ M) compared to ~−24.71 ± 8.7 kcal/mol (K_i_ ~ 1.06 × 10^−18^ M) for ^h^C5a(A8)-C5aR complex, indicating that ^h^C5a(A8) may be a better binder to the modeled C5aR (Table S1). Further, recruitment of the C5aR: N-terminus (1–37) and ECS (38–40, 94–108, 170–201, 261–280) residues, respectively against ^h^C5a: residues (1–74), and ^h^C5a(A8): residues (1–73) provided higher estimated binding free energy of −276 ± 26 kcal/mol for ^h^C5a-C5aR complex and −315 ± 26 kcal/mol for ^h^C5a(A8)-C5aR complex, which clearly indicates the existence of a strong binding interaction between the modeled C5aR and the ligands. However, it is worth mentioning that the estimated binding free energies presented for the complexes are indicative of strong binding only and may not be straightaway translated to experimental binding affinities. Decomposition of the overall binding energy provided the non-bonded interaction energy (summation of van der Waals and electrostatic) for specific “hot-spot” residues, respectively participating at both “site1” and “site2” of ^h^C5a/C5aR and ^h^C5a(A8)/C5aR complexes. The pairwise energy contribution of such “hot-spot” residues for ^h^C5a-C5aR and ^h^C5a(A8)-C5aR complexes are respectively presented in Fig. 7 and Fig. 8. Considering the cationic nature of ^h^C5a^34^, it is clear that the binding at the plausible “site1” is strongly driven by the interaction with a set of anionic residues at the NT, such as D2, D16, D18, D21, and D27 of C5aR. In addition, Y11 and Y14 at the NT of C5aR also contribute significantly toward the binding affinity at the “site1”. This trend is consistent even in case of ^h^C5a(A8)-C5aR complex, where D10, D15, D16, D18, D21 and Y14 make significant contribution toward the binding affinity at the “site1” of C5aR. Further, the K^68^ of ^h^C5a makes significant contribution in contrast to F^67^ of ^h^C5a(A8) at the “site2” of C5aR, matching to our earlier hypothesis that suggests “cation-π” interaction triggers agonism, and “π-π” interaction triggers antagonism at the “site2” of C5aR^9^.

## Discussion

Understanding the molecular basis and conformational dynamics^35–38^ of receptor-ligand interaction is a valuable aspect in the field of pharmacology and drug design^39,40^. While most of the rhodopsin family GPCRs bind to small molecule ligands at a discrete site within the ECS/transmembrane segment^41–43^, there are few GPCRs such as C5aR that bind to small protein ligands like ^h^C5a, which are too bulky to fit completely into the ECS/transmembrane region of C5aR. Thus, early biochemical studies had mapped the high-affinity binding of ^h^C5a to more than one site on C5aR^5,44^, a testable hypothesis that need to be illustrated at an atomistic resolution. Over the years, with advance in structural biology techniques, few GPCRs in complex with small protein ligands have been obtained^6,7,45^ recently, illustrating the idea of “two-site” or multiple site binding models^46–49^. In addition, molecular modeling coupled with biophysical, pharmacological and MD studies have also provided an alternative support to the idea of “two-site” binding models in many GPCRs^29,30,36,50^. Nevertheless, no such highly refined structural complex is currently available for C5aR in the literature. Thus, it remains unclear how ^h^C5a is arrested by the C5aR or what changes does it bring to the C5aR for triggering the activation and downstream signaling.

**Figure 9:**
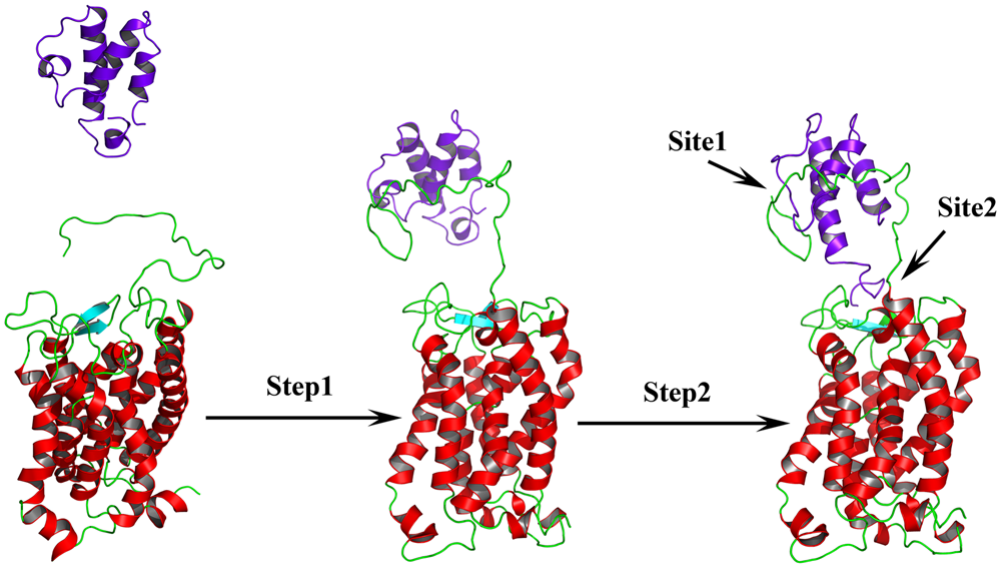
Illustrating the “two-site” binding paradigm of ^h^C5a to C5aR. Step1: Wrapping of NT of C5aR (site1) around the allosteric region of ^h^C5a with high affinity, triggering local conformational change both at the ECS of C5aR and at the CT of ^h^C5a. Step2: Docking of the conformationally altered CT of ^h^C5a at the ECS (site2) of C5aR triggering global conformational change in the overall complex, and further opening of the intracellular face of C5aR for downstream signaling.

In our quest to understand the interaction of ^h^C5a with C5aR better, we recently reported a unique model structure of C5aR in complex with ^h^C5a-CT, PMX53 and NDT^9^. The model complexes indicate that the NT may have a minimal role in binding of small peptide or organic ligands at the “site2” on C5aR, but surely have a potential role in capturing the major part of ^h^C5a. This assumption is well supported by several studies that implicate the role of C5aR NT in high affinity binding of ^h^C5a^18–20,51^, whereas CT peptide of ^h^C5a has been shown to be essential for triggering activation and downstream signaling^52^. Thus, a plausible step wise binding of ^h^C5a to C5aR is hypothesized in this study, which is illustrated in Fig. 9. Briefly, in step-1, the NT (site1) of C5aR wraps around the allosteric region of ^h^C5a^13^ with high affinity, triggering local conformational change both at the ECS of C5aR and at the CT of ^h^C5a. This hypothesis is based on the structure of *des*-Arg^74^-^h^C5a^53^, and ^h^C5a(A8)^10^, whose CT demonstrates an extended β-structure compared to the native ^h^C5a^2^. Interestingly, during the MD simulation over 50 ns^13^, the CT of ^h^C5a also adopts an extended β-structure, deviating from its native α-turn structure. Subsequently in step-2, the conformationally altered CT of ^h^C5a is docked at the ECS (site2) of C5aR, triggering global conformational change in the overall complex, and further opening the intracellular face of C5aR for binding of G-protein or β-arrestin (unpublished data). It is noteworthy that the unique structural illustration of the “two-site” binding in ^h^C5a-C5aR model complex (Fig. 3) finds great support from several studies that interrogated the interaction of ^h^C5a with C5aR by recruiting site-directed mutagenesis studies^15–20,51^. For instance, the high affinity binding at the “site1” of the ^h^C5a-C5aR (Fig. 3) is mainly driven by several salt bridge interactions between D2-H^67^, D21-NH_3_^+^ (M^1^), D27-NH_3_^+^ (M^1^) and hydrogen bond interactions between D16-N^29^, D18-C^27^, D18-R^62^, and T24/T32-M^1^, including several hydrophobic contacts, sustained over 250 ns of MD in POPC bilayer at 300 K (Figs 4 and S7). Literature evidences that both single or (double) mutation of anionic amino acids such as D10N and D27N (D21N, D27N) on NT of C5aR does not affect the binding of ^h^C5a, whereas serial mutations such as (D10N, D15N, D16N) and (D10N, D15N, D16N, D21N, D27N) completely abrogates the binding of ^h^C5a to C5aR^20^. In a separate study, it is also suggested that serial mutations such as (D15A, D16A, D18A, D21A) induces ~42-fold and (D10A, D15A, D16A, D18A, D21A) induces ~140-fold decrease in binding of ^h^C5a^51^, whereas point mutations of D15A, and D18A trigger a remarkable loss in C5aR signaling^19^. Moreover, in the model complex, both Y11 and Y14 also participate in a strong “cation-π” and a strong “π-π” interaction, respectively involving R^37^ and F^51^ of ^h^C5a (Fig. 4). In addition, both Y11 and Y14, including Y6 are also involved in hydrogen bonding, respectively with E^35^, D^24^, and N^64^ of ^h^C5a (Fig. S7). It is evidenced that both Y11 and Y14 undergo sulfation and mutation of Y11F results in complete loss of binding, whereas mutation of Y14F confers ~50% loss in binding affinity of ^h^C5a^18^. Even the low affinity binding of the CT region of ^h^C5a at the “site2” experience numerous interactions with C5aR residues (Figs 4 and S7), whose mutation is known to affect both binding and signaling of ^h^C5a^15–17^. For instance, P184, P270, F275 and N279 of C5aR are involved in strong hydrophobic, hydrogen bonding, and “cation-π” interaction with the K^68^ of ^h^C5a. Similarly, both D191 and S193 are involved in anchoring the R^74^, whereas F182 is involved in a “π-π” interaction with H^67^ of ^h^C5a. Interestingly, the “cation-π” and hydrogen bond interactions of K^68^ with F275 of C5aR remained stable up to 200 ns of MD, whereas the salt bridge and hydrogen bond interactions between R^74^ and D191 of C5aR remained stable up to 170 ns of MD. Its noteworthy that mutation of many such residues of ^h^C5a^54,55^ implicated in the binding of C5aR (Fig. 3b) has been shown to affect both binding and signaling activity of C5aR significantly.

Despite having an altered structure, the engineered antagonist ^h^C5a(A8) also experiences similar level of molecular interactions at both the “site1” and “site2” of C5aR (Fig. 5) with few notable exceptions. The interaction at “site1” involves several hydrogen bonds, salt bridge and hydrophobic interactions that are stable over 250 ns of MD in POPC bilayer and have been summarized, respectively in Fig. 6 and Fig. S14. However, exceptions in interactions are noted at the “site2” of C5aR, where instead of K^68^, F^67^ of ^h^C5a(A8) is engaged in a strong “π-π” interaction with F275 within a cage formed by several strong hydrophobic residues such as F182, P184, and P270 of C5aR (Figs S14 and S8). This differs from the interaction of ^h^C5a at the “site2”, but strongly supports the previously observed interaction of the antagonist PMX53 and inverse agonist NDT at the “site2” of C5aR^9^. Further, this interaction is reversed in case of A8^R69D^ variant of the ^h^C5a(A8), where instead of F^67^, K^68^ is involved in a strong “cation-π” interaction with F275 of C5aR at the “site2”, strongly agreeing with the interactions described for ^h^C5a. Interestingly, mutation of R^69D^ in ^h^C5a(A8) has been described to completely switch the antagonistic action of ^h^C5a(A8) to agonism (IC_50_ ~ 5 nM), at par with ^h^C5a^12^.

**Figure 10:**
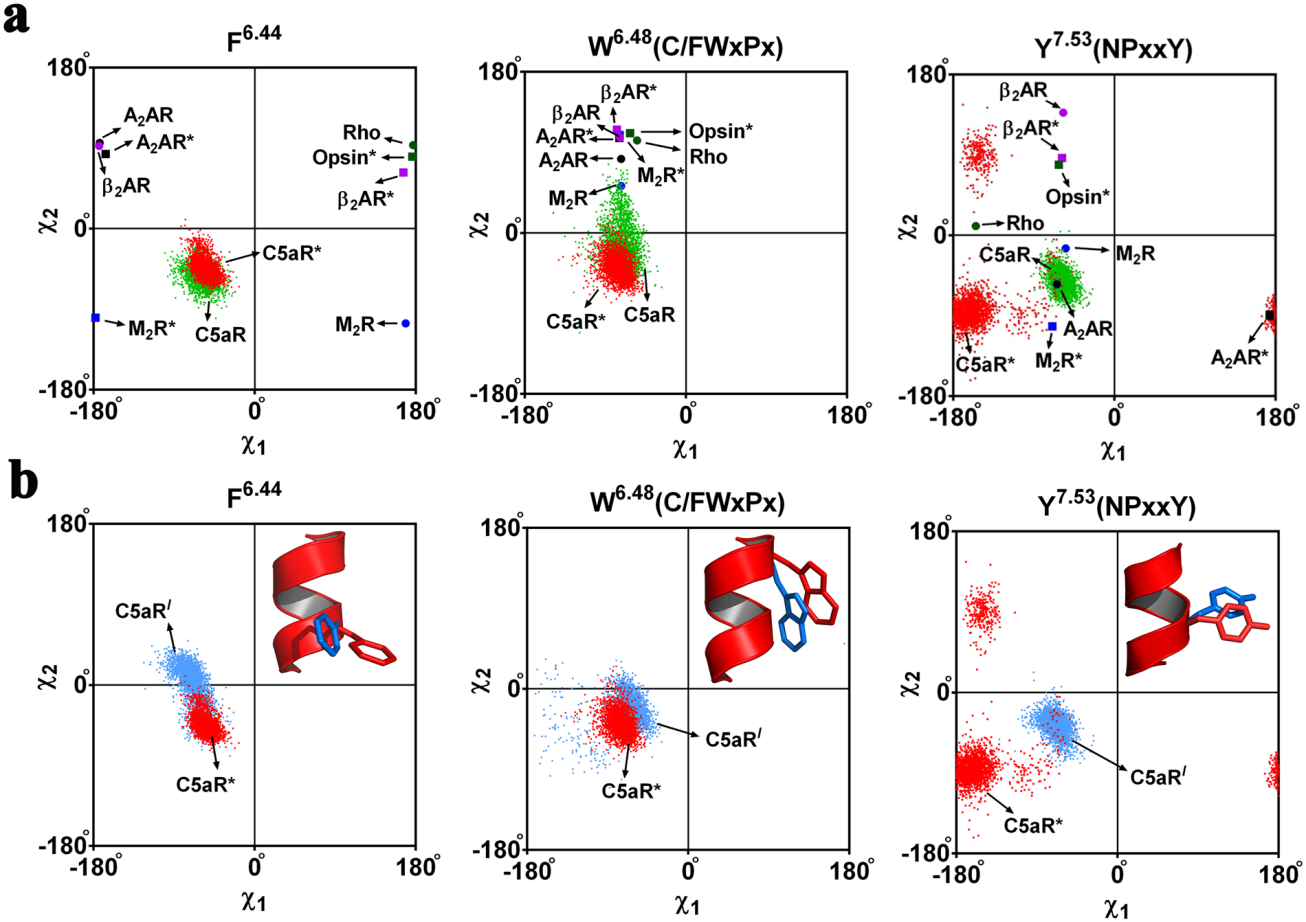
(**a**) Comparative illustration of rotamer toggling (distribution of χ dihedral angles) observed at the conserved regions of C5aR complexed to agonist ^h^C5a and engineered antagonist ^h^C5a(A8) with respect to the other active-inactive pair of GPCRs^41–43,61–65^. (**b**) Comparison of rotamer toggling observed in C5aR on binding to ^h^C5a with respect to free unbound C5aR. Rotamer toggling for F^6.44^, W^6.48^ of “transmission switch” and Y^7.53^ of “tyrosine toggling switch” are illustrated. Active GPCRs are marked in asterisk. C5aR^*I*^: free or unbound C5aR; C5aR: bound to ^h^C5a(A8); C5aR*: bound to ^h^C5a.

Moreover, both the agonist (^h^C5a) and antagonist (^h^C5a(A8) bound complexes illuminate important hallmark information about the activation process of C5aR, in agreement with several rhodopsin family GPCRs reported in the literature^6,7,45^. It is postulated that the breaking of the “ionic lock switch” between R^3.50^ (TM3) and E^6.30^ (TM6) is the hallmark of activation in many rhodopsin family GPCRs^56,57^. In addition, tryptophan (W^6.48^) rotamer toggling at a relatively conserved region (CWxPx) on TM6^58,59^, recently renamed as the “transmission switch” (W^6.48^ and F^6.44^) and the “tyrosine toggle switch” (Y^7.53^) at a conserved region (NPxxY) on TM7 are also known to participate in receptor activation^59^. In our previous studies, we have hypothesized that activation of C5aR in model structures involve movement of almost all the TMs with a higher magnitude of movement noted between TM3 and TM6^8,9^. Since, C5aR lacks an “ionic lock switch”^60^, we shifted our attention to relatively more conserved common activation switches in GPCRs such as the “transmission switch” and the “tyrosine toggle switch” for understanding the effect of ^h^C5a and ^h^C5a(A8) on the model structure of C5aR. As presented in Fig. 10, the concerted rotameric movement of W^6.48^, F^6.44^ (TM6: transmission switch) and Y^7.53^ (TM7: tyrosine toggle switch) at the respective conserved region of C5aR in response to the binding of ^h^C5a (meta-active) and ^h^C5a(A8) (inactive) correlate strongly with the experimental data^49^ of other rhodopsin family GPCRs^41–43,61–65^ (Fig. S16). Further, it appears that transition from inactive to meta-active or pseudo active state in noted GPCRs involve modulation of “π-π” interaction between W^6.48^ and F^6.44^, triggered via swift rotameric transitions (Fig. S16). The extent of “π-π” modulation is directly dependent on the type of neighboring residues. It is interesting to note that unlike the noted GPCRs^41–43,61^, C5aR lacks an “ionic lock switch”^60^, but demonstrates similar trend in rotameric transitions at its conserved region (Figs 10 and S16) on complexation with ^h^C5a and ^h^C5a(A8). Such correlation with experimental observations is surely encouraging, which favorably support the quality of the model complexes presented in the study and further seeds hope that the unique model complexes will withstand the future structural and biophysical scrutiny for further establishing the activation mechanism of C5aR.

## Conclusion

The study provides a partial validation of the unique C5aR model through pilot biophysical studies, illustrating a “two-site” binding interaction of C5aR with two established, contrasting pharmacological counterparts, such as ^h^C5a and ^h^C5a(A8). The presented model complexes illuminate energetically competent inter molecular interactions, largely in sync with reported experimental studies, highlighting the plausible activation mechanism of C5aR. In summary, the model complexes emerge as a significant development in the field for garnering further valuable insights into simple or extended ternary complexes, respectively involving C5aR, ^h^C5a and heterotrimeric G-protein or β-arrestin, which can potentially serve as a template for search and design of disruptive pharmacophores, targeting the chronic inflammation induced malaises.

## Materials and Methods

### Data sets

The NMR structure of ^h^C5a (PDB ID: 1KJS)^2^, crystal structure of ^h^C5a(A8) (PDB ID: 4P39)^10^ and the following rhodopsin family GPCRs (1F88^61^; rhodopsin, 3DQB^62^; opsin*, 2RH1^43^; β2AR, 3P0G^63^; β2AR*, 3REY^42^; A2AR, 3QAK^64^; A2AR*, 3UON^41^; M2R, 4MQS^65^; M2R*) were downloaded from www.rcsb.org. Visualization and presentation of C5aR complexes, including the other GPCRs were performed using DS 4.0 (Accelrys). PyMOL (The PyMOL Molecular Graphics System, Version 1.1r1. Schrödinger, LLC) was used for proper orientation and translation of ligands within the proximity of the extracellular surface (ECS) of C5aR. The starting model of C5aR TM residues were numbered following Ballesteros–Weinstein system^66^. The “cation-π” and “π-π” interaction angles were calculated using our in-house program as described elsewhere^9^. Data were plotted using GraphPad Prism (version 6 for Windows, GraphPad Software, La Jolla California USA, www.graphpad.com).

### Biophysical studies on the ECL2 peptide

The predicted sequence of the ECL2 peptide [Ac-Y174-RVVREEYFPPKVL**C188/S**GVDYSHDKR-R198-NH_2_] of C5aR was prepared using standard Fmoc chemistry over solid phase, by recruiting the services of Genscript (NJ, USA). The analytical HPLC performed over AlltimaTM C18 (4.6 × 250 mm) column, using acetonitrile–water gradient in presence of 0.05–0.065% trifluoroacetic acid (TFA) indicates that the peptide is ≥95% pure (Fig. S1). The integrity of the peptide [Theoretical MW: 3137.52] was confirmed from the presence of the molecular ion peaks at 785.35 for M_H+_ [Observed MW: 3137.40], as observed in ESI-MS. The peptide was completely soluble in water and thus, the secondary structure of 100 µM peptide was analyzed in PBS buffer (pH ~ 7.4) as well as in 100% methanol, by recruiting the JASCO J-815 Circular Dichroism spectropolarimeter. Further, 10–40% of trifluoroethanol (TFE) was added to the PBS buffer to check the effect of TFE on the overall conformation of the peptide. Data were collected at 298 K in 1 cm path length quartz cell with 1 nm bandwidth in Far-UV (200–260 nm) range. Scanning at 50 nm/min with 1.0 s time constant in 1 nm steps, five scans were averaged after baseline correction for solvent. The observations in millidegrees were converted to residue ellipticity [θ_MRW_] with a reported relation^67^. ^1^H-NMR of the peptide was recorded by using a Bruker 800 MHz instrument equipped with a cryoprobe at 298 K in 90% H_2_O/10% D_2_O at pH  ~ 5. Solvent was suppressed using standard Watergate sequence as provided by Bruker.

### Construction of ^h^C5a/^h^C5a(A8) complexes of C5aR

Modeling of C5aR has been extensively detailed in our earlier studies^8,9^. The inactive C5aR (C5aR^*I*^) data and the meta-active C5aR model used in this study has been taken straight from the reported data to build the ^h^C5a-C5aR or ^h^C5a(A8)-C5aR complex further. The starting structure of the NT peptide of the C5aR was obtained from the NMR structure of CHIPS complex (PDB ID: 2K3U)^11^, which was further amino-terminally elongated by adding five amino acids to it using DS 4.0. The modified NT peptide harboring the “site1” was then subjected to flexible automated docking against the central conformer of ^h^C5a populated over 50 ns of MD^13^, by recruiting AutoDock 4.2^68^ and energy minimization by GROMACS in tandem with a carefully designed sequential build-up approach. The best conformer of the NT peptide complexed to the ^h^C5a was subjected to MD over 100 ns at 300 K in presence of explicit water by recruiting the GROMACS package^69^. Further, the most populated conformer of the complex (Fig. S4) bound to the NT peptide of C5aR (site1) was harnessed and the ^h^C5a-CT peptide (^64^NISHKDMQLGR^74^) was removed from the complex. By applying requisite geometrical constraints, the above truncated complex was further subjected to structural assembly with the previously generated central conformer of the major cluster, populated for ^h^C5a-CT complexed to C5aR^9^, resulting the complete ^h^C5a-C5aR complex (Fig. S6). Similar approach was also followed for the construction of ^h^C5a(A8) complex of C5aR. Briefly, the NT of C5aR was docked to ^h^C5a(A8) and continuously refined until no further change in binding energy was noticed (Fig. S11). The CT-peptide of ^h^C5a(A8) and its variants were generated from the parent structure of ^h^C5a(A8) and subsequently subjected to automated docking against the previously described meta-active structure of C5aR^9^. The C5aR complexed to the CT-peptide variants of ^h^C5a(A8) at the “site2” were subjected to MD in POPC bilayer over 100 ns each. The most populated conformers of the major clusters were respectively harnessed (Fig. S10) and further subjected to structural assembly with the bulk of ^h^C5a(A8) complexed to NT of C5aR, resulting the complete ^h^C5a(A8)-C5aR complex (Fig. S13).

### Molecular dynamics studies

The ^h^C5a-C5aR and ^h^C5a(A8)-C5aR complexes were inserted into POPC [1-palmitoyl-2-oleoyl-sn-glycero-3-phosphocholine] bilayer using InflateGRO^70^ and further subjected to independent MD simulations for 250 ns each at 300 K by recruiting the GROMACS package^69^, as described previously^8,9^. Both ^h^C5a-C5aR and ^h^C5a(A8)-C5aR systems were charge neutralized by randomly placing 19 and 21 chloride ions respectively, in presence of 16724 and 16586 water molecules. Both the systems were equilibrated twice, first for 5 ns under NVT, followed by 50 ns under NPT conditions prior to the MD studies. Conformational clustering was performed as described with a time interval of 20 ps^8^. The utility programs available in GROMACS were implemented for detailed analysis of all the MD trajectories.

### Estimation of binding free energy

Molecular mechanics Poisson-Boltzmann surface area (MM-PBSA) method, as implemented in g_mmpbsa program^71^ was used for calculating the binding free energies of both ^h^C5a-C5aR and ^h^C5a(A8)-C5aR complexes. Briefly, the binding free energies were estimated using implicit water by Poisson Boltzmann (PB) approaches. The solvation energy of the solute was estimated by using a dielectric continuum to account for the electrostatic as well as the non-polar contribution. The dielectric constant for the solvent and the solute were taken as 80 and 20 respectively for polar calculation. The non-polar contribution to solvation free energy term was calculated from solvent-accessible surface area (SASA). The grid spacing was set to 0.5 Å. Probe radius for SASA estimation was set to 1.4 Å. 150 conformers, randomly selected each from the first major cluster, populated for ^h^C5a-C5aR (Fig. S15a) and ^h^C5a(A8)-C5aR (Fig. S15b) complexes, respectively over 250 ns of MD in lipid bilayer were subjected to MM-PBSA based binding free energy calculation, by recruiting the interacting residues only, as illustrated, respectively in Fig. 3b (^h^C5a-C5aR) and Fig. 5b (^h^C5a(A8)-C5aR). MM-PBSA and the energy contribution of individual amino acids toward overall binding free energy of the complex were respectively calculated by utilizing the “MmPbSaStat.py”, and “MmPbSaDecomp.py” scripts.

## Structural Note

While this manuscript was under peer review, the crystal structure of a thermostabilized C5aR with 11 mutations both in TMs and in Loops (StaR; PDB ID: 5O9H) bound to an inverse agonist appeared in the literature^72^. On comparison, it was noted that the StaR shares an incredible structural similarity with our previously modelled C5aR^8^ (backbone RMSD ~ 5 Å), which is used for generating the current structural complex. Our truncated model structure of the native C5aR, respectively lacked 26 residues on N-terminus and 34 residues on C-terminus. Interestingly, the StaR also lacks 29 residues from the N-terminus and 17 residues from the C-terminus. More importantly, we had hypothesized that the ECL2 peptide [Ac-Y174-RVVREEYFPPKVLCGVDYSHDKR-R198-NH_2_] could be an independently folded unit, which is most likely to adopt a β-hairpin like conformation in solution, as also evidenced in the current study. Indeed, as predicted, the StaR illustrates a β-hairpin structure exactly in the same region. Further, our inactive model C5aR illustrated TM1: P36-F64 (StaR: V35-A66); TM2: F75-Q98 (StaR: I69-Q98); TM3: I111-F139 (StaR: G106-F139); TM4: W154-F172 (StaR: A150-Y174); TM5: E199-R228 (StaR: R197-S231); TM6: L241-V260 (StaR: K239-S266); TM7: F275-V302 (StaR: T274-A303) and TM8: F307-L315 (StaR: P316-L323), which is in excellent agreement with the experimentally derived model structure of StaR. In addition, we had even observed a single α-turn in the ICL2 of our modelled C5aR, in contrast to the two-turn α-helical structure observed in StaR, which collectively provides a direct evidence supporting the excellent quality of our model.

## Electronic supplementary material

Supplementary Information
