# Supplementary material for: The Model Structures of the Complement Component 5a Receptor (C5aR) Bound to the Native and Engineered hC5a
Source: Sci Rep. 2018 Feb 13;8:2955. doi: 10.1038/s41598-018-21290-4 (PMC5811428; doi:10.1038/s41598-018-21290-4)
Supplement: Supplementary file 1 — Supplementary Information [file 41598_2018_21290_MOESM1_ESM.pdf]

Electronic Supplemental Information

The Model Structures of the Complement Component 5a Receptor (C5aR) Bound to the Native and Engineered ^{h}C5a.

Amita Rani Sahoo[1], Richa Mishra[1], and Soumendra Rana*[1]

[1]*Chemical Biology Laboratory, School of Basic Sciences, Indian Institute of Technology Bhubaneswar, Bhubaneswar, Odisha 752050, India. Email: soumendra@iitbbs.ac.in*

Supplemental Figures S1 to S16 and Table S1 are provided.

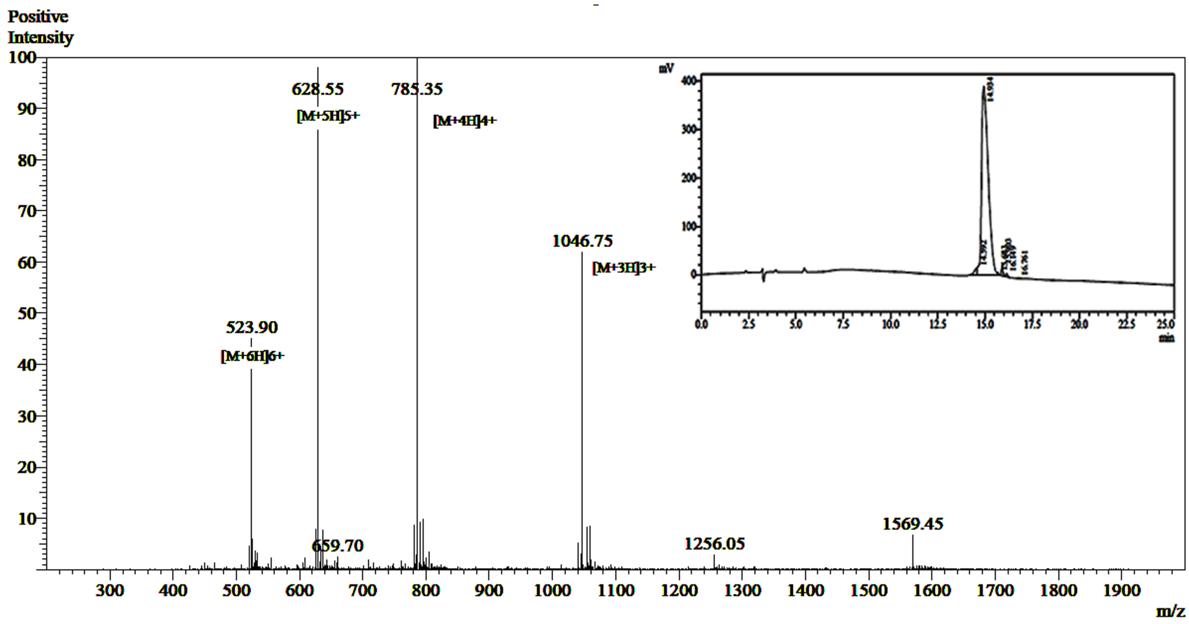

Figure S1. The ESI-MS ($MW_{theoretical}$=3137.52, $MW_{observed}$=3137.40) and analytical HPLC profile (inset) of the ECL2 peptide (>95% purity) of C5aR.

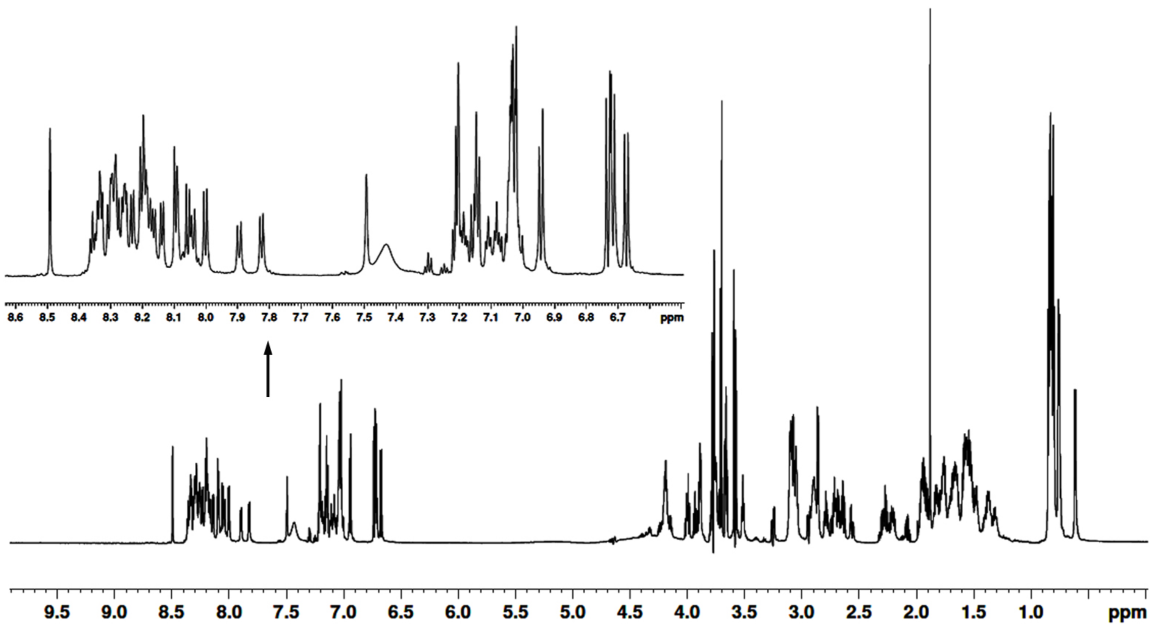

Figure S2. ^{1}H NMR spectra of the ECL2 peptide of C5aR (~ 0.6 mM, 800 MHz) recorded at 298K in 90% water-10% D_2O solution of pH ~ 5.

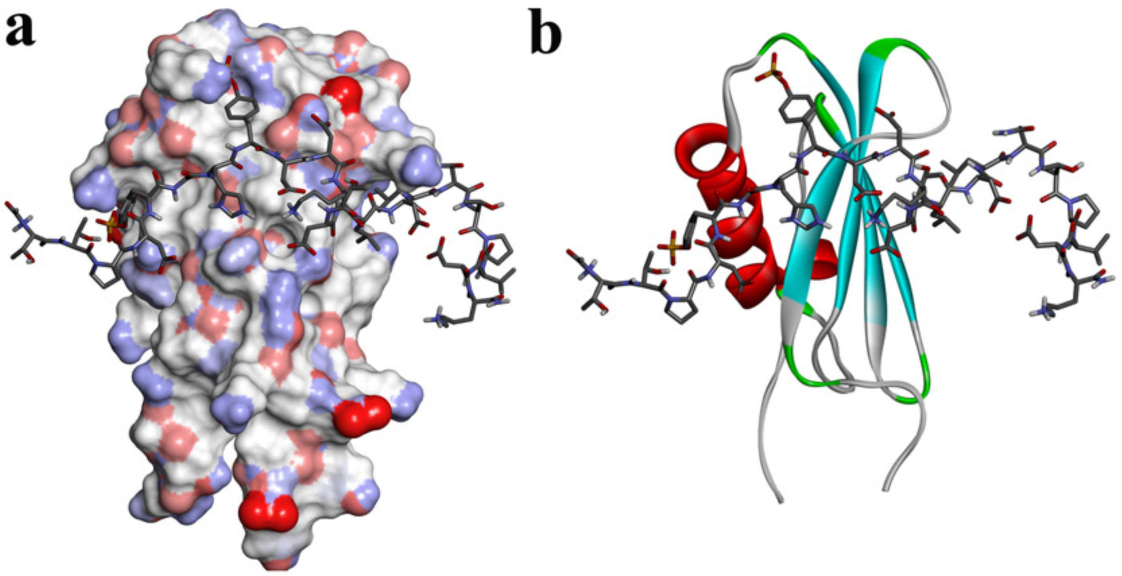

Figure S3. (a) Solution NMR structure of CHIPS protein bound to NT peptide of C5aR (PDB ID: 2K3U). Molecular surface of CHIPS protein shown in ionizability mode, highlighting the bound conformer of the NT peptide of C5aR. (b). Cartoon representation of CHIPS protein docked against the NT peptide of C5aR (Ki ~ 674 µM; ~ -4.33 kcal/mol). Backbone RMSD between the docked and original conformer of NT peptide is 0.001 Å.

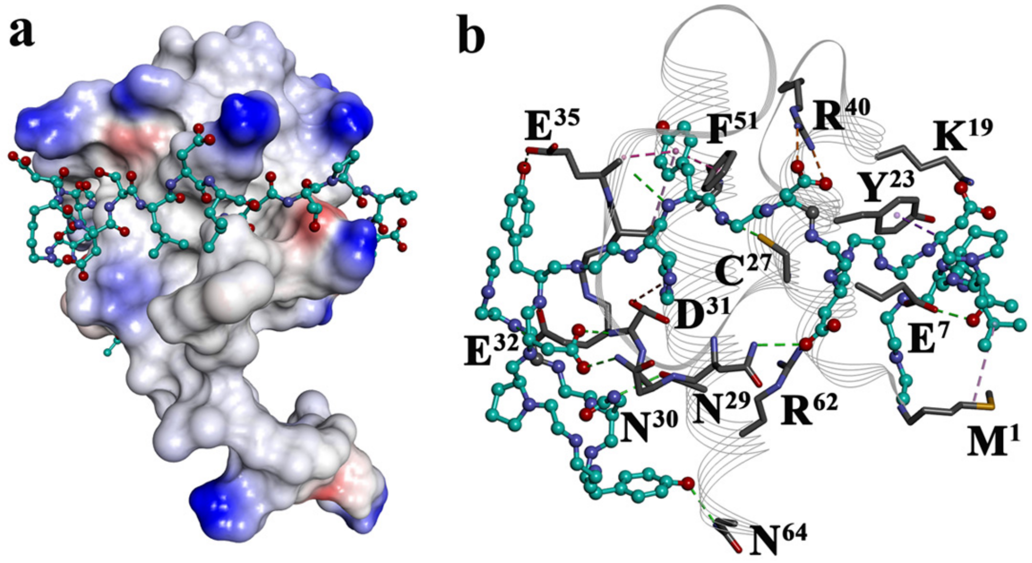

Figure S4. (a) The NT peptide of the C5aR harbouring the "site1" complexed to the major conformer of ^{h}C5a evolved over 50 ns of MD. (b) Specific inter molecular interactions between the ^{h}C5a and "site1" of C5aR (cyan). ^{h}C5a residues are numbered. Hydrogen bond, electrostatic and hydrophobic interactions are respectively shown as green, orange and purple dashed lines.

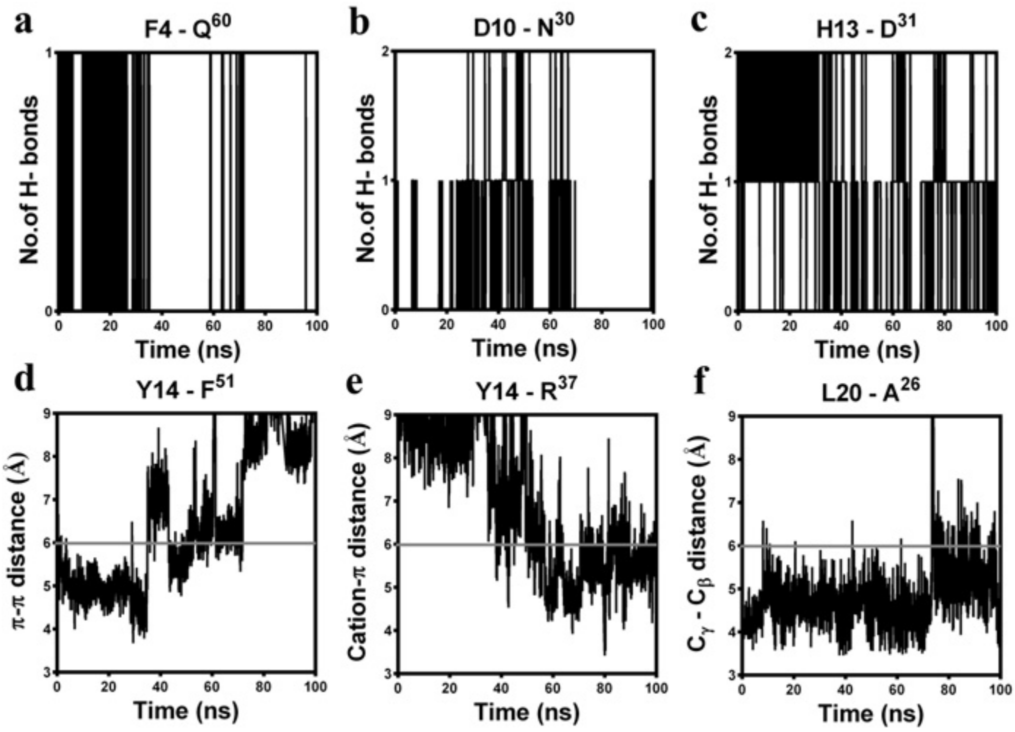

Figure S5. Monitoring the stability of some intermolecular interactions observed between ^{h}C5a and NT peptide of C5aR, over 100 ns of MD at 300K. The superscripts indicate that the interacting

residue is part of the ^{h}C5a. The solid grey lines indicate the cut-off distance. (a) Stable hydrogen bonding observed between the backbone CO of F4 and side chain of Q^{60}. (b) Moderate hydrogen bonding between the side chains of D10 and N^{30}. (c) Stable hydrogen bonding between the side chains of H13 and D^{31}. (d) Stable "π-π" interaction observed between Y14 and F^{51} up to 70 ns. (e) Strong "cation-π" interaction between Y14 and R^{37} evolved after 40 ns of MD. (f) Strong hydrophobic interaction observed between Cγ of L20 and Cβ of A^{26}.

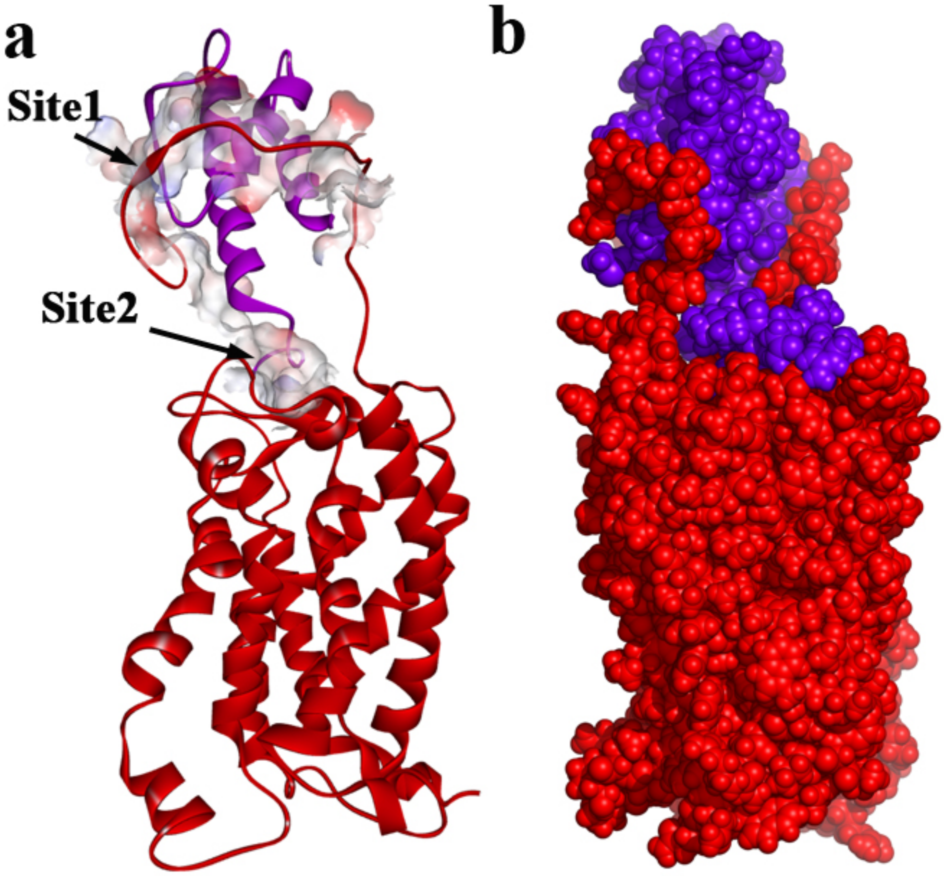

Figure S6. (a) Cartoon representation of the ^{h}C5a-C5aR model structural complex, respectively highlighting the "site1" and "site2" at the N-terminus and the ECS of C5aR. (b) CPK model displaying binding of ^{h}C5a (violet) to the C5aR (red).

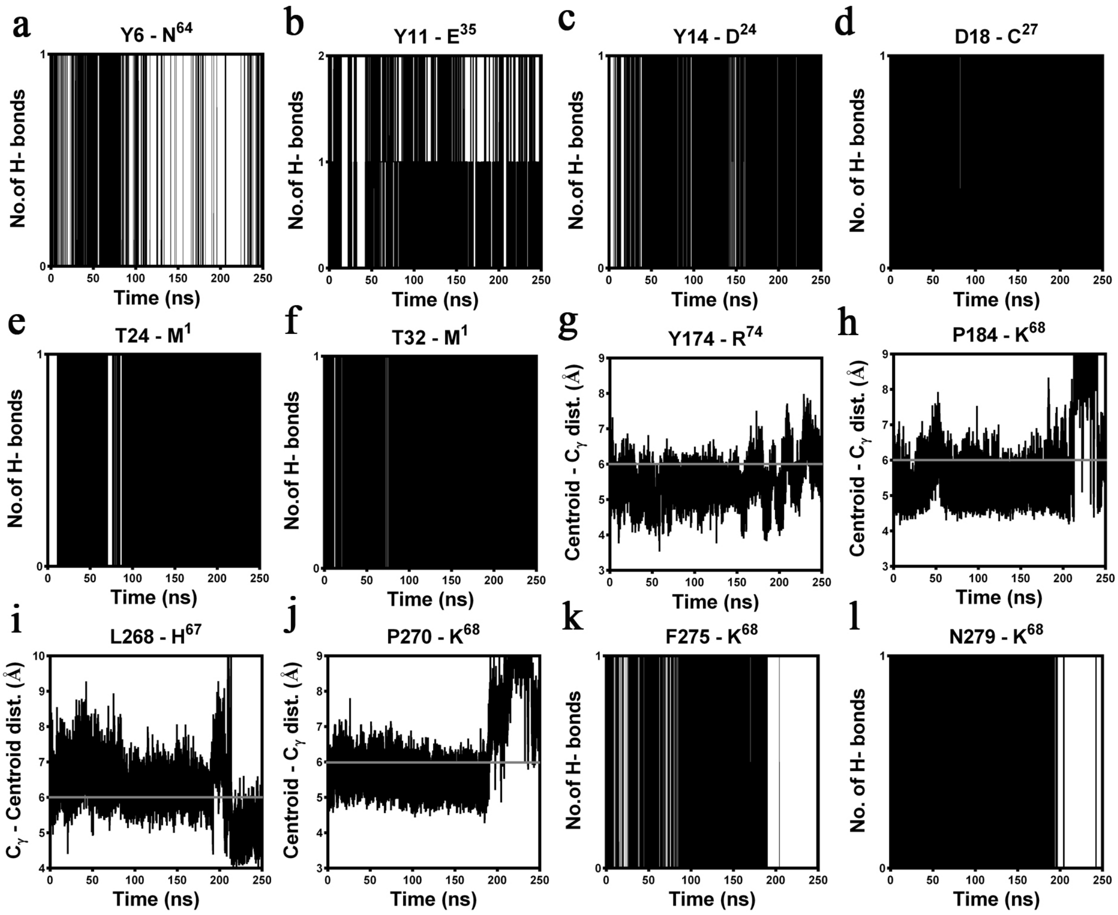

Figure S7. Monitoring the stability of intermolecular interactions between the "hot-spot" residues of the ^{h}C5a-C5aR complex over 250 ns of MD in POPC bilayer. The superscripts indicate that the interacting residue is part of the ^{h}C5a. The solid grey lines indicate the cut-off distance. (a) Stable hydrogen bond interaction observed between the side chains of Y6 and N^{64}. (b) Strong hydrogen bond interaction between the side chains of Y11 and E^{35}. (c) Stable hydrogen bond interaction between side chain of Y14 and backbone CO of D^{24}. (d) Very strong hydrogen bond interaction observed between backbone NH of D18 and backbone CO of C^{27}. (e) Stable hydrogen bond interaction between backbone CO of T24 and the NH_3^+ terminus of M^1. (f) Strong hydrogen bond interaction between backbone CO of T32 and the NH_3^+ terminus of M^1. (g) Hydrophobic interaction between the centroid of Y174 and Cγ of R^{74}. (h) Hydrophobic interaction between the centroid of P184 and Cγ of K^{68}. (i) Strong hydrophobic interaction between the Cγ of L268 and centroid of H^{67}. (j) Hydrophobic interaction between the centroid of P270 and Cγ of K^{68}. (k) Stable hydrogen bond interaction observed up to 200 ns between the backbone CO of F275 and the head group of K^{68}. (l)

Strong hydrogen bond interaction between the side chain of N279 and the head group of K^{68} is observed up to 200 ns of MD.

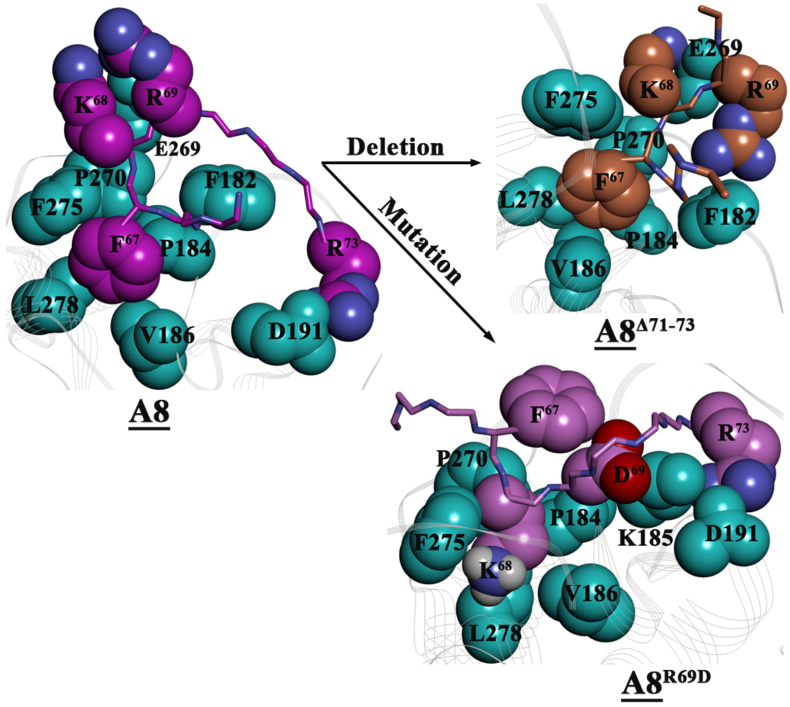

Figure S8. Illustration of the comparative binding modes of CT-peptides of ^{h}C5a(A8) (purple), $A8^{\Delta 71\text{-}73}$ (brown) and $A8^{R69D}$ (light pink) at the "site2" of C5aR (cyan). "Site2" is occupied by F^{67} and surrounded by a cluster of hydrophobic residues in both ^{h}C5a(A8), and $A8^{\Delta 71\text{-}73}$ complexes. But, in case of $A8^{R69D}$, the "site2" is occupied by K^{68}.

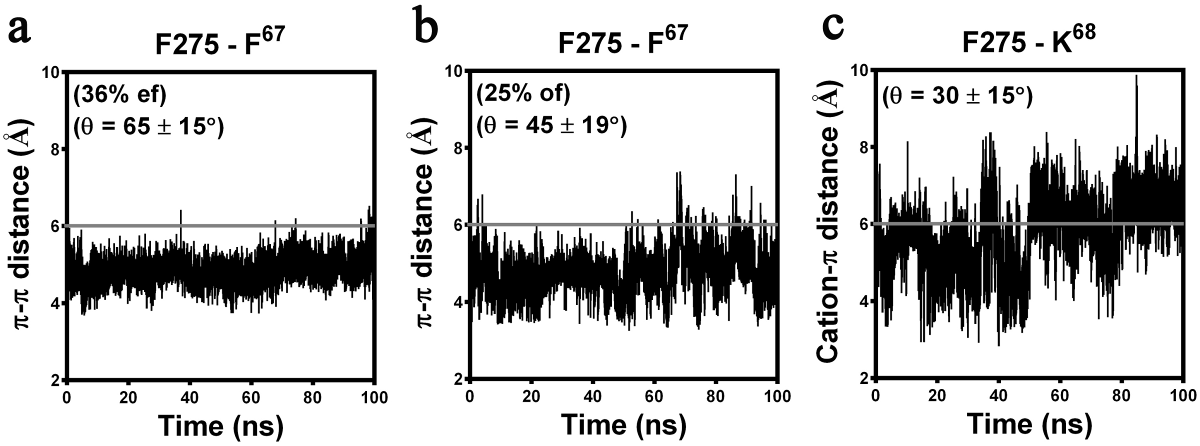

Figure S9. Comparison of distinct intermolecular interactions observed for the CT peptide variants of ^{h}C5a(A8) at the "site2" of C5aR over 100 ns of MD in POPC bilayer. (a) Illustration of strong "π-

π" interaction (*ef*) observed between the aromatic rings of F275 of C5aR and F^{67} of the CT peptide of ^{h}C5a(A8). (b) Illustration of strong "π-π" interaction (*of*) observed between the aromatic rings of F275 of C5aR and F^{67} of the CT peptide of A8$^{\Delta 71\text{-}73}$. (c) Illustration of stable "cation-π" interaction monitored between the aromatic ring of F275 and the head group of K^{68} of the CT peptide of A8^{R69D}.

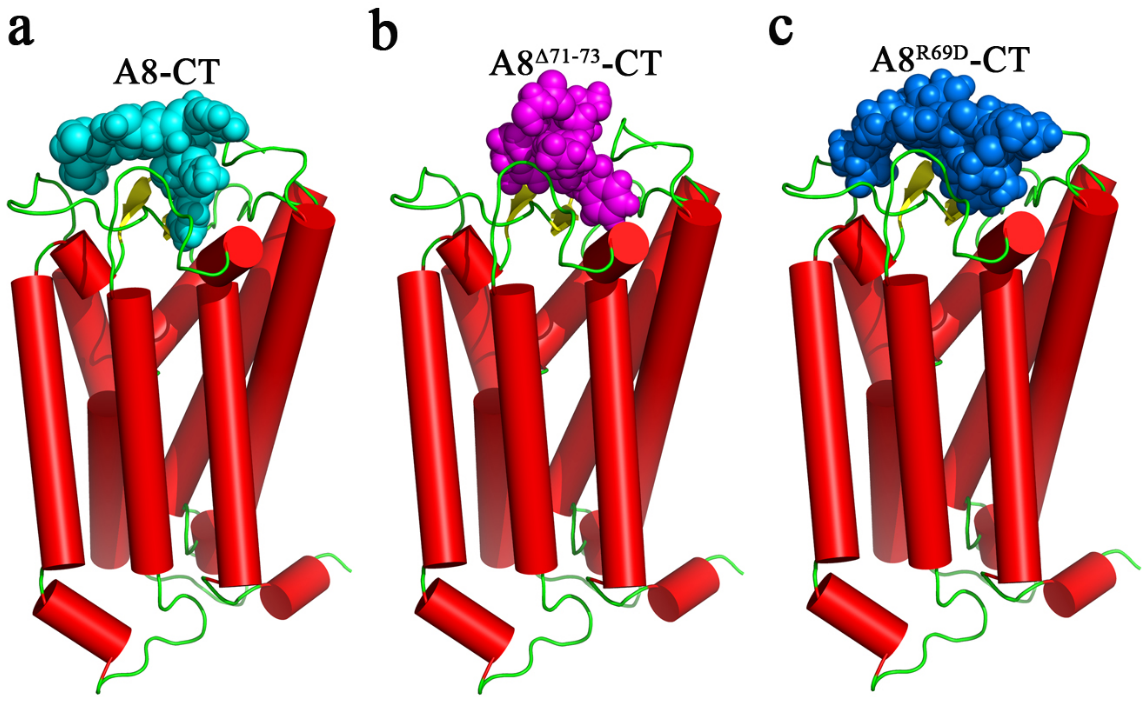

Figure S10. Comparative illustration of the major conformer of C5aR (red) respectively complexed with CT peptide variants of ^{h}C5a(A8) populated over 100 ns of MD in POPC bilayer. (a) The C5aR complexed to the CT peptide (^{64}NISFKRSLLR^{73}) of ^{h}C5a(A8) highlighted in cyan. (b) The C5aR complexed to the CT peptide (^{64}NISFKRS^{70}) of A8$^{\Delta 71\text{-}73}$ highlighted in magenta. (c) The C5aR complexed to the CT peptide (^{64}NISFK**<u>D</u>**SLLR^{73}) of A8^{R69D} highlighted in blue.

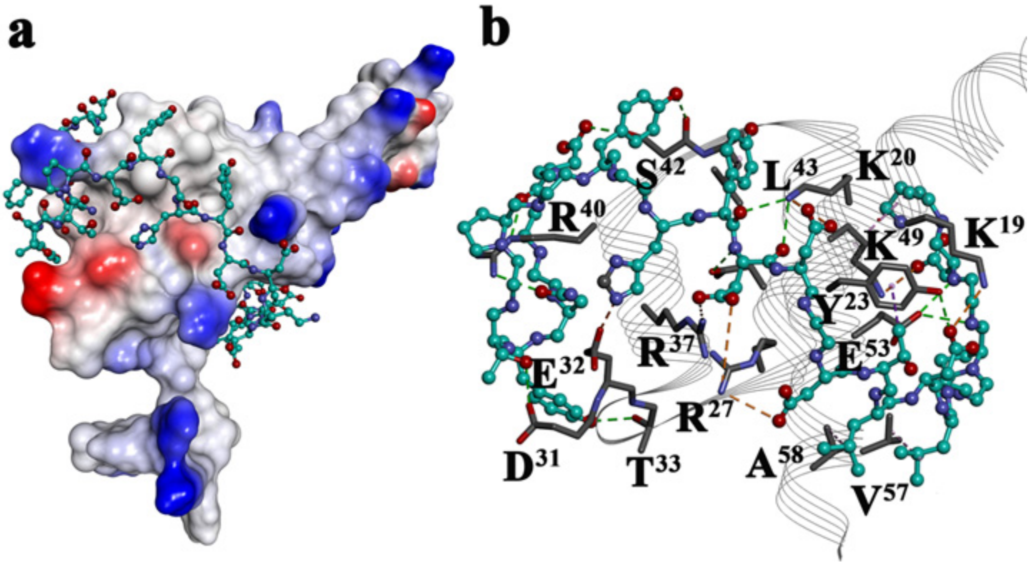

Figure S11. (a) The NT peptide of the C5aR harbouring the "site1" complexed to the major conformer of ^{h}C5a(A8), evolved over 50 ns of MD at 300K. (b) Specific inter molecular interactions between the ^{h}C5a(A8) and "site1" of C5aR (cyan). ^{h}C5a(A8) residues are numbered. Hydrogen bond, electrostatic and hydrophobic interactions are respectively shown as green, orange and purple dashed lines.

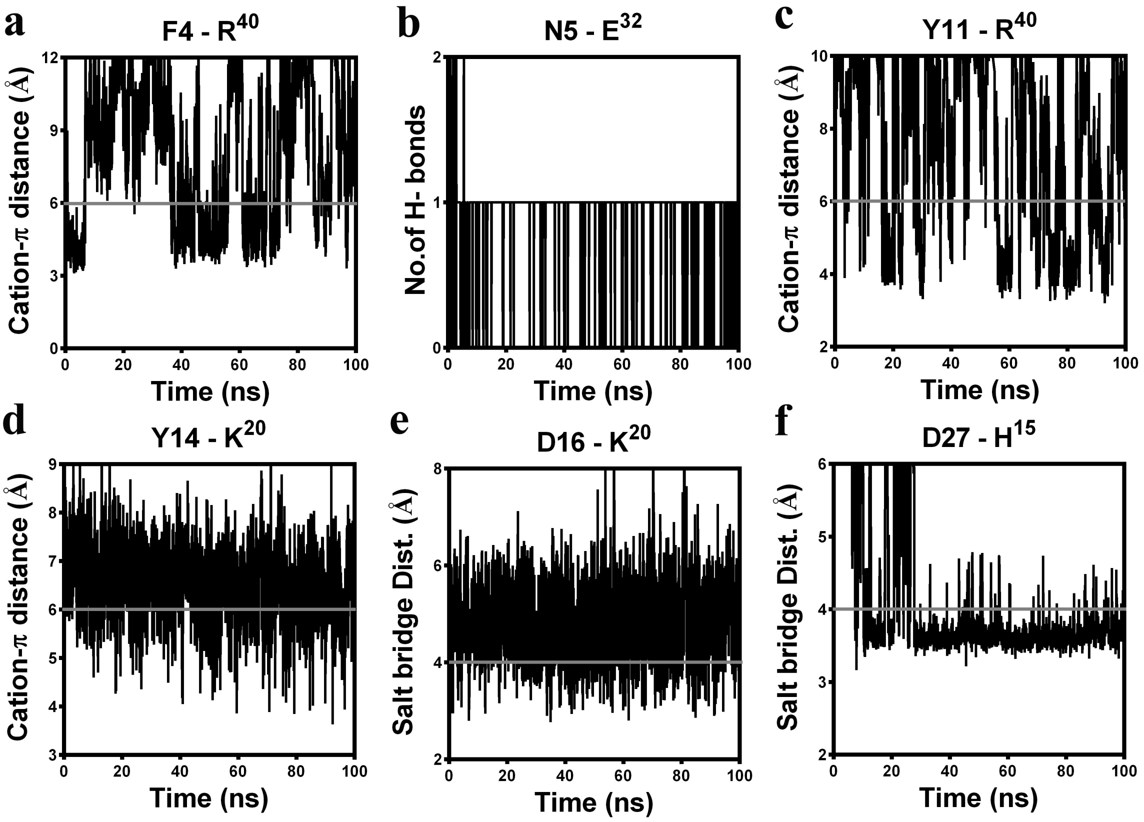

Figure S12. Monitoring the stability of some intermolecular interactions between ^{h}C5a(A8) and NT of C5aR over 100 ns of MD. The superscripts indicate that the interacting residue is part of the ^{h}C5a(A8). The solid grey lines indicate the cut-off distance. (a) Moderate “cation-π” interaction between F4 and R^{40} is observed. (b) Stable hydrogen bond interaction between side chains of N5 and E^{32}. (c) Strong “cation-π” interaction is observed between Y11 and R^{40}. (d) Strong “cation-π” interaction is observed between Y14 and K^{20}. (e) Strong salt bridge interaction observed between D16 and K^{20}. (f) Strong salt bridge interaction observed between D27 and H^{15}.

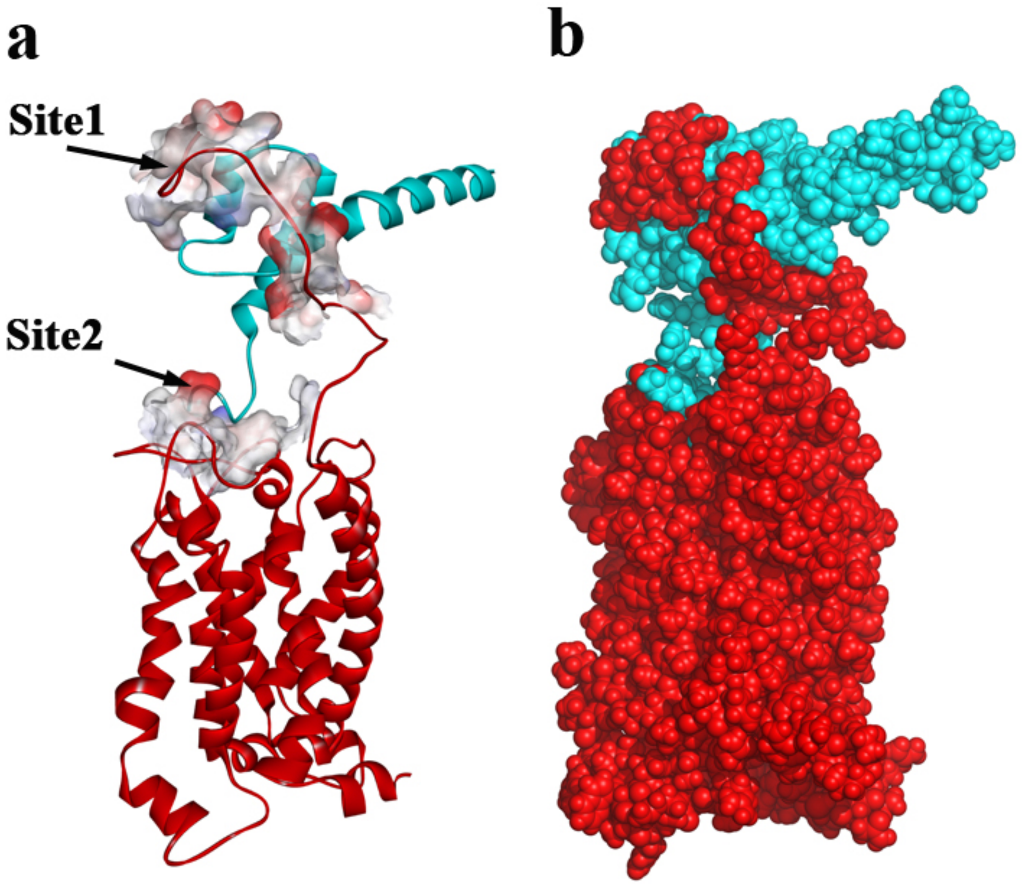

Figure S13. (a) Cartoon representation of the ^{h}C5a(A8)-C5aR model structural complex, respectively highlighting the “site1” and “site2” at the N-terminus and the ECS of C5aR. (b) CPK model displaying binding of ^{h}C5a(A8) (cyan) to the C5aR (red).

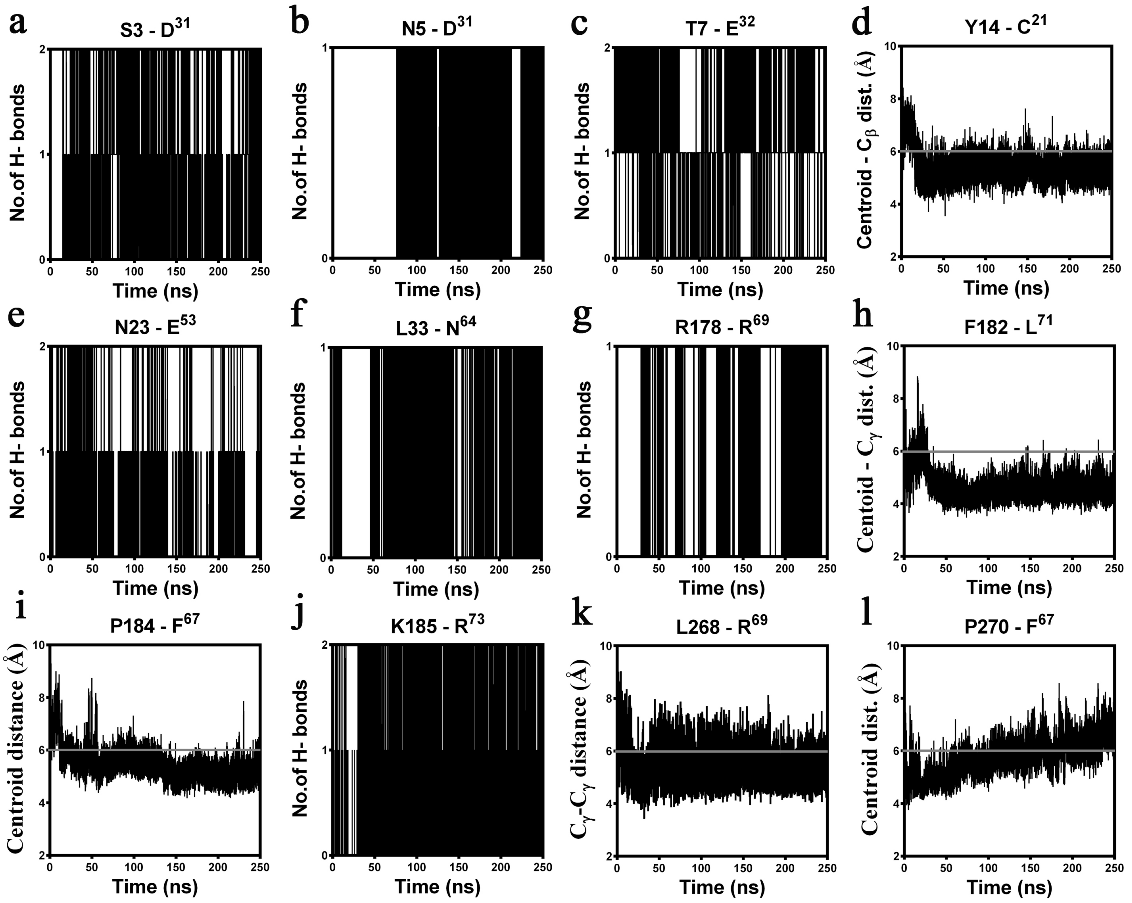

Figure S14. Summary of the sustained intermolecular interactions observed between the "hot-spot" residues at the "site1" and "site2" of the ^{h}C5a(A8)-C5aR complex over 250 ns of MD studies in POPC bilayer. The superscripts indicate that the interacting residue is part of the ^{h}C5a(A8). The solid grey lines indicate the cut-off distance. (a) Stable hydrogen bond interaction observed between the side chains of S3 and D^{31}. (b) Strong hydrogen bond interaction observed between the side chain of N5 and the backbone NH of D^{31}. (c) Hydrophobic interaction observed between the centroid of Y14 and Cβ of C^{21}. (d) Very strong hydrogen bond interaction observed backbone NH of D18 and backbone CO of C^{27}. (e) Stable hydrogen bond interaction observed between the side chains of N23 and E^{53} (f) Strong hydrogen bond interaction observed backbone NH of L33 and backbone CO of N^{64}. (g) Moderate hydrogen bond interaction observed between the side chain of R178 and backbone CO of R^{69} (h) Hydrophobic interaction between the centroid of F182 and Cγ of L^{71}. (i) Strong hydrophobic interaction between the centroid rings of P184 and F^{67}. (j) Very strong hydrogen bond observed between the head group of K185 and the CO_2^- terminus of R^{73}. (k) Strong hydrophobic

interaction between Cγ atoms of L268 and R[69]. (l) Hydrophobic interaction observed between the centroid rings of P270 and F[67].

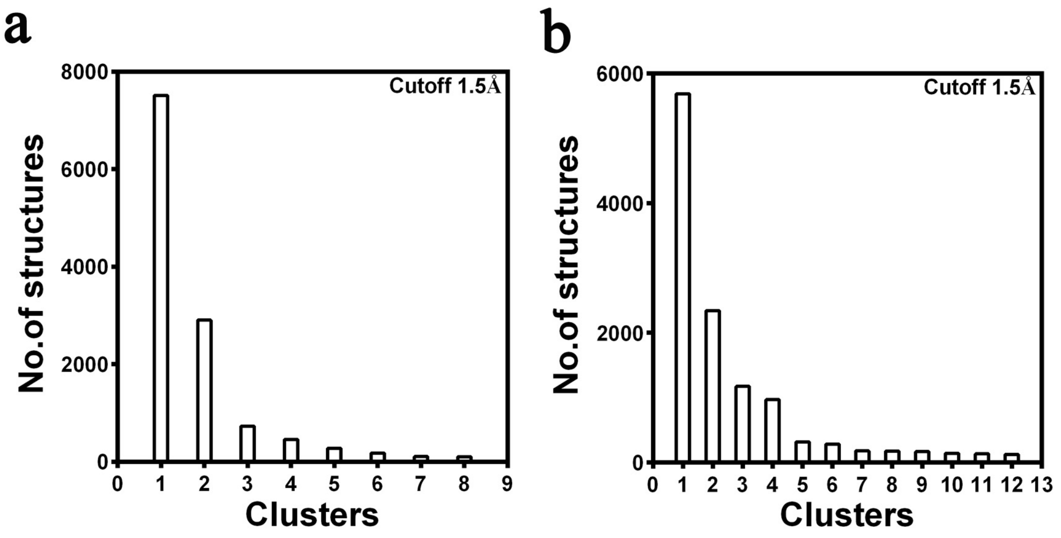

Figure S15. Cluster analysis displaying the major conformational microstates, respectively populated for (a) ^{h}C5a-C5aR and (b) ^{h}C5a(A8)-C5aR complexes over 250 ns of MD in POPC bilayer.

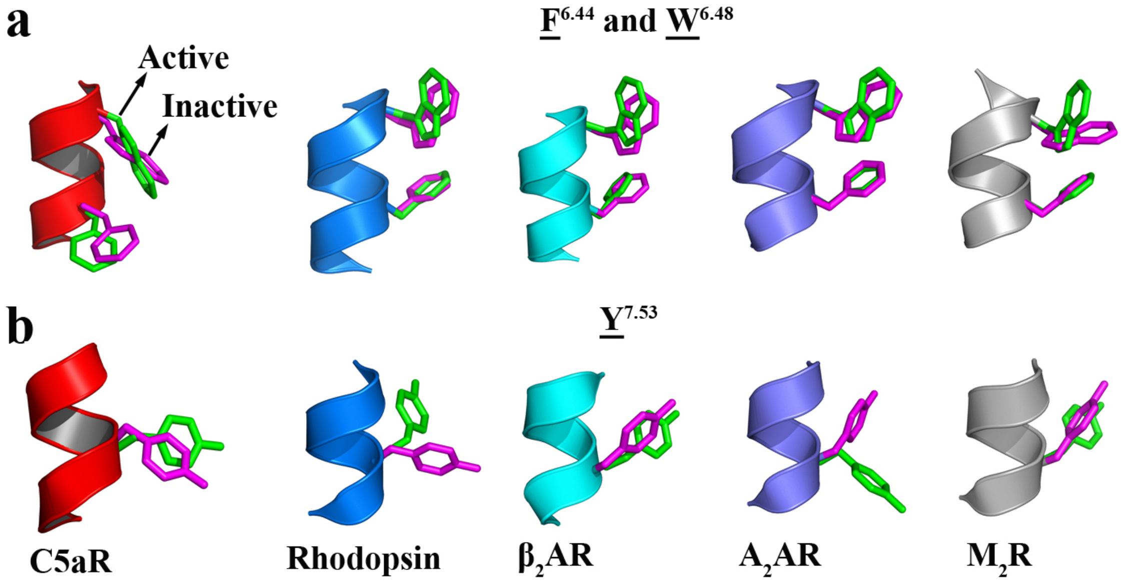

Figure S16. Comparative illustration of rotamer toggling observed at the conserved regions of model C5aR complexes in reference to the rhodopsin family of GPCRs. Rotamers of specific residues in active (green) and inactive (magenta) GPCRs are highlighted in sticks. (a) Change in rotameric states of $F^{6.44}$ and $W^{6.48}$ residues of "transmission switch" on TM6 observed in C5aR along with other

known GPCRs. (b) Rotameric transition of $Y^{7.53}$ residue of "tyrosine toggle switch" on TM7 of conserved NPxxY motif.

Table S1. Comparison of average binding free energy between the ^{h}C5a-C5aR and ^{h}C5a(A8)-C5aR complex, estimated over 150 conformers, each isolated from the 1st cluster, respectively populated over 250 ns of MD in POPC bilayer.

**Energetic Contribution**	**^{h}C5a-C5aR* Complex (kcal/mol)**	**^{h}C5a(A8)-C5aR* Complex (kcal/mol)**
van der Waal energy	-173.8 ± 12.8	-187 ± 8.9
Electrostatic energy	-75.2 ± 4.2	-36.4 ± 2.06
Polar Solvation Energy	254.5 ± 16.2	219.8 ± 11.2
SASA energy	-21.5 ± 1.2	-21.08 ± 0.88
Total Binding Energy	-16.12 ± 4.2	-24.71 ± 8.7

* The interacting residues taken for MM-PBSA calculation are depicted in Fig. 3 and Fig. 5.
